# Dynamic pre-structuration of lipid nanodomain-segregating remorin proteins

**DOI:** 10.1038/s42003-024-07330-y

**Published:** 2024-12-05

**Authors:** Zeren Xu, Adrien Schahl, Marie-Dominique Jolivet, Anthony Legrand, Axelle Grélard, Mélanie Berbon, Estelle Morvan, Louis Lagardere, Jean-Philip Piquemal, Antoine Loquet, Véronique Germain, Matthieu Chavent, Sébastien Mongrand, Birgit Habenstein

**Affiliations:** 1 Univ. Bordeaux, CNRS, Bordeaux INP, CBMN, UMR 5248, IECB, F-33600 Pessac, France; 2 Institut de Pharmacologie et de Biologie Structurale, Université de Toulouse, CNRS, Université Paul Sabatier, 31400 Toulouse, France; 3 Sorbonne Université, LCT, UMR7616 CNRS,75005Paris, France; Qubit Pharmaceuticals, Advanced Research Department, 75014 Paris, France; 4 Laboratoire de Biogenèse Membranaire (LBM) UMR-5200, CNRS-Univ. Bordeaux, F-33140 Villenave d’Ornon, France; 5 Univ. Bordeaux, CNRS, Inserm, IECB, UAR3033, US01 Pessac, France; 6 Laboratoire de Microbiologie et Génétique Moléculaires (LMGM), Centre de Biologie Intégrative (CBI), Université de Toulouse, CNRS, UPS, Toulouse, France

**Keywords:** Computational biophysics, Plant molecular biology, Supramolecular assembly, Protein folding

## Abstract

Remorins are multifunctional proteins, regulating immunity, development and symbiosis in plants. When associating to the membrane, remorins sequester specific lipids into functional membrane nanodomains. The multigenic protein family contains six groups, classified upon their protein-domain composition. Membrane targeting of remorins occurs independently from the secretory pathway. Instead, they are directed into different nanodomains depending on their phylogenetic group. All family members contain a C-terminal membrane anchor and a homo-oligomerization domain, flanked by an intrinsically disordered region of variable length at the N-terminal end. We here combined molecular imaging, NMR spectroscopy, protein structure calculations and advanced molecular dynamics simulation to unveil a stable pre-structuration of coiled-coil dimers as nanodomain-targeting units, containing a tunable fuzzy coat and a bar code-like positive surface charge before membrane association. Our data suggest that remorins fold in the cytosol with the N-terminal disordered region as a structural ensemble around a dimeric anti-parallel coiled-coil core containing a symmetric interface motif reminiscent of a hydrophobic Leucine zipper. The domain geometry, the charge distribution in the coiled-coil remorins and the differences in structures and dynamics between C-terminal lipid anchors of the remorin groups provide a selective platform for phospholipid binding when encountering the membrane surface.

## Abstract (web summary)

NMR structure calculations combined with molecular dynamics simulations suggest a conserved pre-structuration into coiled-coil dimers of the plant protein remorin before associating to the membrane.

## Introduction

Cellular functions related to plasma membrane (PM)-binding proteins often require tight and dynamic regulation because of their cellular localization at the communicating interface between the cytoplasm and the environment. First discovered in tomato and potato, remorin (REM), a plant PM-binding protein, controls numerous signaling pathways in immunity, symbiosis, and development^1,2^. A prominent example of REM-dependent functions is the regulation of plasmodesmata closure during immune responses such as viral infection or exposure to salicylic acid^3,4^.

REM variants compose a multigenic family containing six subgroups, specific to the land-plant lineage^5^. Containing three common major architectural components, REMs are capable of homo-multimerization^6–8^, membrane association^1,7,9,10^, lipid nanodomain segregation, and site-specific cellular localization^11^. Classified upon their aminoacid motif composition in the N-terminal intrinsically disordered region (IDR), the REMs compose a phylogenetic family containing 6 groups. In principle, the cellular localization of REMs from distant groups can coincide, such as for group 1 and 6 REMs, which partially target the plasmodesmata at the plasma membrane in *Oryza sativa* and in *Solanaceae*^3,12^.

Yet, the REMs of different groups perform a panoply of distinct functional activities in plant cells^5,11^, are tightly regulated, and selectively localized in separate nanodomains, mainly excluding members of evolutionary distant REM groups^13,14^.

The architecture of the family members, comprising three domains, includes a C-terminal anchor (REM-CA) responsible for dynamic membrane anchoring. For *St*REM1.3, the REM-CA confers anionic lipid specificity during membrane association^9,10,15^. In particular, REM1.3 is selective for sterol-, PIP- and anionic phospholipids- containing membranes, forming 70-90 nm wide nanodomains in vivo and in vitro^3,15–17^ with increased membrane order and thickness^1,9,10^. The REM-CA of several REM groups contains a Cysteine (Cys) residue susceptible to S-acylation, such as the S-palmitoylation previously observed for remorins^18–20^. Neighboring the REM-CA, remorins contain a coiled-coil domain (CC) that assembles into high molecular weight filaments with the specific signature of all-α-helical protein components^6,7^. When perturbing the oligomerization by introducing Leucine (Leu, L) mutations in this domain of *St*REM1.3 to Proline (Pro, P) or Glutamate (Glu, E), suggested to perturb the intermolecular interface in the coiled-coil domain, membrane association is impaired^7,10^. At the N-terminal ending, an IDR of variable length confers sites for protein–protein interactions and regulation by post-translational modifications such as phosphorylation^11^ for REMs of all phylogenetic groups. Phosphorylation-mimicking mutations in the N-terminal domain modulate the membrane nanodomain clustering in vitro and in vivo, suggesting a regulative role of the electrostatic properties in the IDR, fine-tuning lipid-protein segregation on the membrane^1,10^. Thus, while each domain is associated with specific functions for some members of the REM family, this can neither be translated to the whole family nor rationalized.

We here aim at deciphering and reasoning the molecular determinants that confer REMs the remarkable capacity of precisely performing diverse functions, accurately targeting distinct cellular localizations and selectively clustering into lipid- and protein-enriched nanodomains. We choose an experimental in vivo and in vitro approach, including confocal and total internal reflection fluorescence (TIRF) microscopy as well as NMR spectroscopy, combined with extensive analysis by bioinformatics and molecular simulations to identify the structural domains, the structural and dynamic determinants of the protein domains and their interplay that modulate remorin’s properties to associate to membrane nanodomains.

## Results

### Architecture and conservation in the remorin protein family

**Fig. 1:**
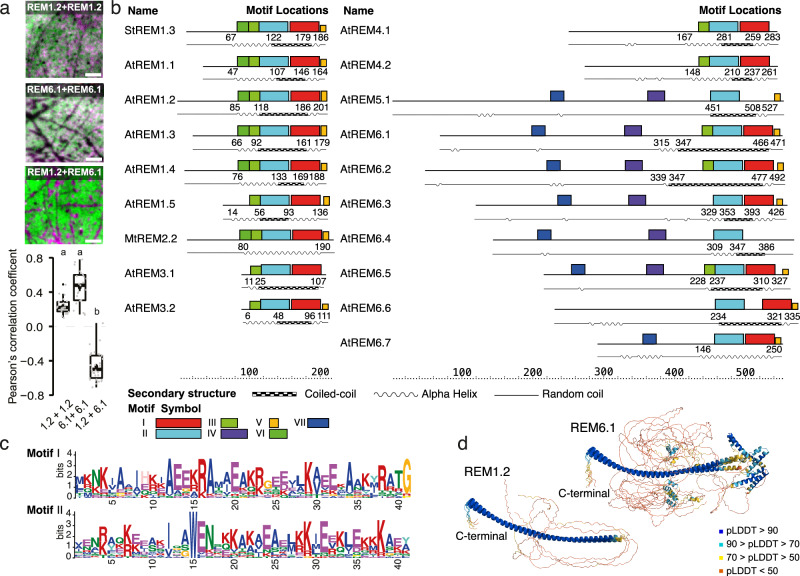
In vivo and in silico analysis of remorin architecture. **a** Representative dual-color TIRF images of the surface of epidermal cells of *Nicotiana benthamiana* transiently co-expressing full-length AtREM1.2 and/or AtREM6.1, labeled with mRFP1.2 (magenta) or mVenus (green). AtREM1.2/AtREM1.2 and AtREM6.1/AtREM6.1 co-localize, while AtREM1.2 (magenta) and AtREM6.1 (green) are mutually exclusive. The Pearson’s correlation coefficient of REM1.2 and/or REM6.1, shown in the lower panel, was calculated from at least 23 cells over the course of three independent experiments. Significant differences were determined using a Kruskal–Wallis test followed by a Dunn’s multiple comparison test. Different letters indicate significant differences (*p* > 0.01). Scale bar = 4 μm. **b** Representation of 19 remorin homologs indicating (1) sequence motifs as determined by MEME^22^ (motif symbols are shown below), (2) a cartoon of the structure prediction by Alphafold2 in ColabFold (AF2)^23,24^, (3) regions in coiled-coil conformation predicted by Multicoil2^25,26^ (checkerboard boxes). **c** Primary sequence of motifs 1 and 2, located in the predicted coiled-coil region. **d** Secondary structure prediction by AF2 is shown for an ensemble of 5 structures, colored by predicted local distance difference test (pLDDT)^23^ score.

Earlier work suggested that REMs assemble into filamentous structures and contain CC domain^6,7,21^, associate with lipids on the membrane surface^9,10,15^, cluster into nanodomains^9,10,14,15,18^ and locate into different nanodomains dependent on their phylogenetic group^14^. To reason the differential localization for REMs of different groups, we first used dual-color Total Internal Reflection Fluorescence microscopy (TIRF) on *Nicotiana benthamiana* leaves expressing a chosen REM pair from different groups, namely remorin of group 1 (REM1.2) and/or 6 (REM6.1), respectively labeled with an mRFP1.2 or mVenus tag for the combinations REM1.2/1.2, REM6.1/6.1, REM1.2/6.1. As expected^14^, proteins from the same group (REM1.2/1.2, REM6.1/6.1) tend to co-localize as represented by the Pearson’s coefficient and the microscopic pattern in Fig. 1a, whereas REM1.2/6.1 cluster in clearly separated nanodomains (Material and method, M&M, subsection: Cloning, Plant culture and protein expression, Dual-color TIRF microscopy, Statistics and reproducibility). To distinguish primary structural elements potentially responsible for the differential localization we then chose REMs of all phylogenetic groups to determine in a comparative approach their amino acid and structural motif composition, as detected by the MEME suite^22^, correlated with the Alphafold2 in ColabFold (AF2)^23,24^ structure prediction and the coiled-coil domain limits given by Multicoil2^25,26^, presented in Fig. 1b (M&M subsection: Sequence alignment and structure prediction, Coiled-Coil Prediction Using Multicoil2, Motif Prediction Using MEME). Globally, 5 motifs, located in the region predicted in an α-helical conformation by AF2, and in the C-terminal anchor, are highly conserved over the different family groups with slight variations between the group members (Fig. 1b and Supplementary Fig. 1a). The groups displaying large N-terminal regions, mostly predicted as IDR, contain two additional motifs of which one has an α-helical conformational propensity detected by AF2. However, AF2 tends to overestimate the propensity of proteins to form alpha helices^27^. The C-terminal-specific motif 5, located in the anchor REM-CA (Fig. 1b, colored orange), is represented in most REM group members. This motif contains highly conserved hydrophobic residues such as Phenylalanine (Phe) and acylation-prone Cysteines (Supplementary Fig. 1a), as well as less conserved hydrophobic Tryptophane (Trp) and Alanine (Ala), confirming that membrane association relying on an unconventional C-terminal anchor structural motif, such as proposed for *St*REM1.3^15^, is conserved in the REM family. Sequence alignment (Supplementary Fig. 1b) demonstrates that protein groups, in which motif 5 is not detected (AtREM3.1, 4.1, 4.2 and 6.4), still harbor the conserved hydrophobic or potentially acylated residues. Furthermore, all groups contain one or more conserved prolines, that allow to break α-helix secondary structures, and glycines (Gly) promoting secondary structure kinks or breaks. These sequence components indicate potential selective insertion or association of specific protein segments to the membrane and to the lipid headgroups as previously suggested for the C-terminal anchor of StREM1.3^9,10,15^. The neighboring residues consequently contain several positively charged lysine (Lys) and Arginine (Arg) residues conferring the specificity to negatively charged lipid headgroups such as the PI4P for *St*REM1.3^9,10^. The distribution of the positively charged Arg and Lys within and surrounding motif 5 varies among the groups, possibly modulating the structural trapping of presented lipid headgroups at the membrane surface. In all chosen family members, motif 1 and motif 2 are partially present (Fig. 1b, c and Supplementary Fig. 1b), both overlapping with predicted α-helical conformation and coiled-coil quaternary structure. Both motifs contain numerous conserved positively charged Arg, Lys, and negatively charged Glutamate (Glu) and Aspartate (Asp) as well as conserved hydrophobic residues. Motifs III and VI (in light and dark green) are partially conserved over the REM family members and contain mostly hydrophobic and charged conserved residues (Fig. 1b and Supplementary Fig. 1a). AF2 predicts the helical structure encompassing the above-mentioned motifs with high confidence (Fig. 1b, d and Supplementary Fig. 2). The N-terminal region is mainly predicted as intrinsically disordered, which is coherent with the previously suggested structural features of this domain^5,9,28^. However, two motifs indicated in blue in Fig. 1b, d have been predicted in this region whereof the most N-terminal positioned motif shows a propensity for α-helical conformation.

### Variations in structure and dynamics of the C-terminal membrane anchor

**Fig. 2:**
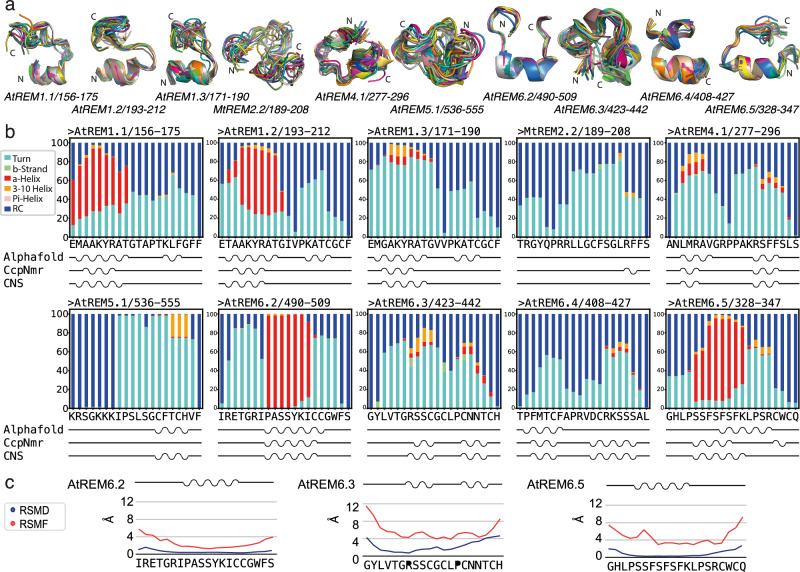
Structures and dynamics of the remorin C-terminal anchor (REM-CA) in solution. **a** Structural bundle of ten NMR structures. N- and C-terminal endings are indicated on the structures. **b** Secondary structure propensity of REM-CA structures simulated with atomistic molecular dynamics simulations using the AMOEBA forcefield^30^ over 1 μs. The color code for structural motifs is indicated aside (RC random coil). Indicated below is a cartoon of the secondary structure as determined by AF2 performed on the respective REM-CA, CcpNMR analysis^31^, and CNS structure calculation^29^. **c** Examples for the RMSD of the NMR structures and the RMSF of MD simulations are shown for the REM-CA of AtREM6.2, AtREM6.3, and AtREM6.5 (complete analysis is provided in Supplementary Fig. 4 and Supplementary Videos).

Because the primary sequence of REM-CA varies between the different REM groups and membrane association depends on the anchoring of this segment to the presented lipids at the surface, we followed the hypothesis that the structure of the anchor might significantly impact REM behavior during membrane anchoring. We, therefore, decided to delineate whether REM-CA dynamically pre-structures before encountering the membrane surface. We chose a 20-residue segment, including the motif V, to compare the structural conformation of ten selected representatives of the REM family groups in the solution (Supplementary Fig. 3). The REM-CA selection followed several criteria, including the intention of having one representative per main phylogenetic group, a high primary sequence divergence, and including a higher number of REM-CA representative from two distant groups, which have been demonstrated to not co-localize in vivo, i.e. group 1 and 6, with REM-CA in group 6 being very diverse in primary sequence^14^. Multi-dimensional NMR spectroscopy allowed to sequentially assign the NMR resonances (Supplementary Fig. 3) and derive structural restraints from inter-residual atomic proximities (Supplementary Data 1) (M&M subsection: Peptide synthesis and specification, peptide sample preparation for NMR, NMR experiments and analysis, structure calculation). Based on the experimental NMR restraints, we calculated structural ensembles of the REM-CA segments, using the CNS algorithm^29^, presented in Fig. 2a. While the backbone root mean square deviation R.M.S.D. of the ten structures varies between the individual peptides, all REM-CA peptides adopt a partially α-helical fold towards the N-terminal ending neighboring the coiled-coil domain. In all structures, a kink is observed similar to the kink that we have previously proposed to occur on the membrane surface, partially inserted into the membrane bilayer in StREM1.3^15^. The kinks are centered around a conserved Gly, recognized as terminal residue in motif 1 (Figs. 1c, 2a), and/or Pro residue in all peptides, which occurs in one or two repetitions in all groups, mostly in a GxxP pair, while its location is not conserved (Supplementary Fig. 1b).

Because the structural ensembles and the backbone R.M.S.D. of the NMR structures indicate different underlying mobility rather than distinct structures of the REM-CA peptides, we used atomistic molecular dynamics (MD) to simulate the peptide dynamics based on different force fields (M&M subsection: Molecular dynamics simulation using Amber fields, Molecular dynamics simulation using the polarizable force field AMOEBA and Adaptive sampling). Figure 2b compares the distribution of the secondary structure adopted by each residue of the peptide sequence over the MD simulation of 1 μs of Adaptive Sampling MD simulations using the AMOEBA forcefield^30^ (Supplementary Videos) using a calculated NMR structure as template, the AF2 secondary structure prediction, the secondary structure proposed by CcpNmr Analysis, based on the chemical shifts^31^, and the calculated structure using CNS^29^. Adaptive sampling MD simulations using the AMOEBA forcefield have the advantage to, respectively, optimally sample the conformational space through sequentially sampling the previously calculated conformational space, and to most accurately represent atomic charges and polarity with respect to other available forcefields^32^ (M&M subsection: Molecular dynamics simulation using the polarizable force field AMOEBA and Adaptive sampling). These data show that α-helical regions observed in the NMR-based structures correlate well with secondary structure propensities observed by the MD simulation of 1 μs for the peptides in which the helical segments encompass >6 residues. Beyond the secondary structure, the peptide dynamics can be accurately estimated by combining MD simulations and NMR. The NMR-derived RMSD, representing the different static structures calculated based on NMR restraints, and MD root mean square fluctuation (RMSF), quantifying the fluctuation of the residue over the simulation period, are compared for representatives of REM-CA structures. Represented structures reflect tendencies towards lower RMSD and RMSF (AtREM6.2), higher RMSD and RMSF (AtREM6.3) and lower RMSD and higher RMSF (AtREM6.5) in Fig. 2c (complete analysis is provided in Supplementary Fig. 4a). As expected, RMSD values globally correlate with RMSF values, higher RMSF are observed in sections with higher RMSD. The RMSD in the α-helical regions structural segments is comparatively low, as observed for AtREM1.1, AtREM1.2, AtREM6.2, and AtREM6.5. In structures with high RMSD values, such as AtREM2.2, AtREM4.1, and AtREM6.3 the structural motifs are unfolding during MD simulations, reflecting a more important dynamics in these peptides. Note that discrepancies between the secondary structure content of the determined structure, coherent with the automatic detection by the CcpNMR suite, and the AF2 predictions occur often in segments with high RMSD but also in a few cases for short fragments with low RMSD. To test the impact of the force field used in the MD simulations, we performed the simulation implementing two common force fields, namely Amber ff14sb and Amber ff99sb (M&M subsection: Molecular dynamics simulation using Amber fields). While Amber ff14sb provided better results than Amber ff99sb when compared to the structural motifs observed by NMR, the AMOEBA force field yielded overall the most comparable results to the experimental data (Supplementary Fig. 4b). This indicates that the polar interactions contributing to the structure of REM-CA peptides are best represented by the AMOEBA forcefield, coherent with the presence of a significant number of charged and polar amino acids within the stably structured motifs (Fig. 1b).

**Fig. 3:**
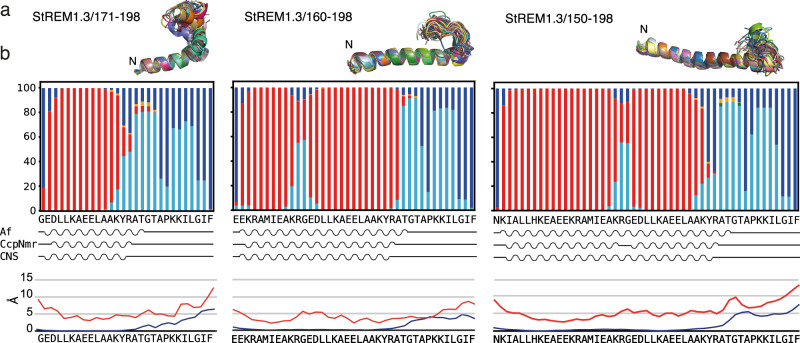
Structures and dynamics of the extended remorin C-terminal anchor (REM-CA) in solution. **a** Structural bundles of ten NMR structures for StREM1.3_171-198_, StREM_160-198_, and StREM_150-198_. The N-terminal ending is indicated on the structures. **b** Structure propensity of REM-CA structures simulated with atomistic molecular dynamics simulations using the AMOEBA forcefield^30^ over 1 μs (see also Supplementary Videos). The color code for structural motifs is as in Fig. 2b. Indicated below is a cartoon of the secondary structure as determined by AF2 performed on the respective REM-CA, CcpNMR analysis^31^, and CNS structure calculation^29^, as well as the RMSD (blue) of the NMR structures and the RMSF (red) of MD simulations.

Because the α-helical secondary structure predicted by AF2 extends through the primary sequence of the CC domain towards the REM-CA peptides (Fig. 1d and Supplementary Fig. 2), we tested which conformations link the α-helical domain to the REM-CA for selected StREM1.3 constructs. We assigned the NMR chemical shifts and performed NMR data-based structure calculation of longer C-terminal constructs, namely StREM1.3_171-198_, StREM1.3_160-198_, and StREM1.3_150-198_ (Fig. 3a and Supplementary Fig. 5). The determined structures (StREM1.3_171-198_ PDB ID 9F1E, StREM1.3_160-198_ PDB ID 9F1F, and StREM1.3_150-198_ PDB ID 9F1G, Table 1) reveal that the short α-helical secondary structure in REM-CA extends toward the N-terminal α-helical CC domain but tends to form a kink through a Gly residue (Fig. 3a). This suggests that REM-CAs adopt an α-helical fold that connects with the potential coiled-coil domain through an extended helix, containing a slight kink around the Gly residue, highly conserved in groups 1, 2, and 4.

**Table 1:** Refinement statistics for NMR structure calculation

Protein	St13 150–198	St13 160–198	St13 171–198
**NMR distance and dihedral constraints**			
Distance constraints	1057	876	634
Total NOE	1057	876	634
Intra-residue	496	389	283
Inter-residue	561	487	351
Sequential (|i – j| = 1)	254	198	130
Medium-range (|i – j| < 4)	293	266	195
Long-range (|i – j| > 5)	14	23	26
Intermolecular	0	0	0
Hydrogen bonds	0	0	0
Total dihedral angle restraints	53	44	27
*ϕ*	28	22	14
*ψ*	25	22	13
**Structure statistics**			
**Violations (mean and s.d.)**			
Distance constraints (Å)	0.050 ± 0.011	0.052 ± 0.021	0.044 ± 0.016
Dihedral angle constraints (°)	1.670 ± 0.386	0.815 ± 0.471	1.919 ± 0.378
Max. dihedral angle violation (°)	2.302	2.002	2.223
Max. distance constraint violation (Å)	0.300	0.360	0.280
**Deviations from idealized geometry**			
Bond lengths (Å)	0.013 ± 0.0001	0.013 ± 0.0002	0.015 ± 0.0003
Bond angles (°)	1.14 ± 0.020	1.16 ± 0.035	1.19 ± 0.031
Impropers (°)	0.98 ± 0.013	1.07 ± 0.072	1.15 ± 0.052
Average pairwise r.m.s. deviation*	A151–188,A190–193	A161–185,A187,A191	A173–186,A190–194,A197
Heavy (Å)	1.09 ± 0.29	1.16 ± 0.30	1.01 ± 0.20
Backbone (Å)	0.74 ± 0.31	0.75 ± 0.37	0.66 ± 0.18

^*^Pairwise RMSD was calculated among ten refined structures.

To test the dynamics in StREM1.3_171-198_, StREM1.3_160-198_, and StREM1.3_150-198_, we used one deposited structure of StREM1.3_171-198_ (PDB ID 9F1E), StREM1.3_160-198_ (PDB ID 9F1F), and StREM1.3_150-198_ (PDB ID 9F1G) and performed MD simulations as described above, using adaptive sampling MD simulations based on the AMOEBA forcefield (M&M subsection: Molecular dynamics simulation using the polarizable force field AMOEBA and Adaptive sampling). The structures stably maintained their conformations over 1 μs MD simulations, without losing structural integrity, including the flexible kink fold around the Gly residue (Fig. 3b). MD simulations, therefore, corroborate structure calculations based on NMR data, indicating that the kink in StREM1.3, notably present in the longest construct REM1.3_150-198_ is a pre-structured motif with increased flexibility as indicated by the higher RMSF in this segment (mainly KRG). Again, AMOEBA yielded more coherent results than Amber ff99sb. Results obtained using Amber ff14sb and AMOEBA are similar, however, using ff14sb, the slight kink of increased flexibility is placed in a distinct location for StREM_160-198_, in which NMR does not detect structural deviations from the helix, lost in the longest construct and the simulations are less consistent with the NMR-based structure in the terminal regions of the longer constructs (Supplementary Fig. 6, M & M subsection: Molecular dynamics simulation using Amber fields).

Our combined data on structures and dynamics from NMR and MD simulations indicate that the C-terminal endings, anchoring REMs to the lipid headgroups, stably pre-structure in solution with specific structures for all groups before encountering the membrane surface. Structural elements are distributed in rather comparable patterns within the group members and differ between the groups, indicating differences in the mode of action during lipid association between groups of the REM family. These observations are supported by the potential of several REM-CAs for S-acylation, containing up to four Cys residues in the anchor region, that could significantly enhance membrane-anchoring propensity^19,20^. The pre-structuration of the C-terminal anchor should provide a motif with high specificity and the desired affinity for the targeted membrane of specifically lipid-enriched membrane regions.

### Differential localization into nanodomains depends on the C-terminal region

**Fig. 4:**
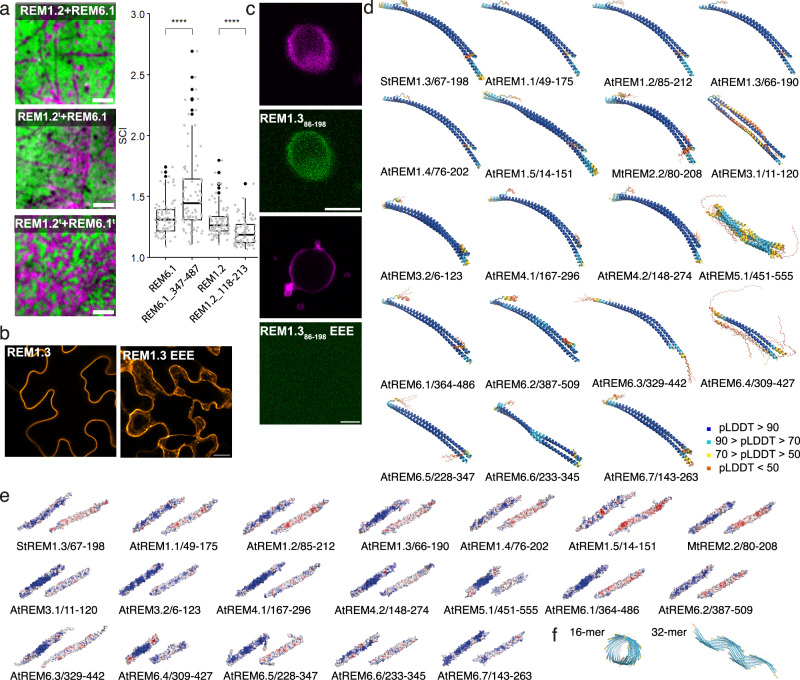
In vivo, in vitro, and in silico analysis of remorin architecture. **a** Representative dual-color TIRF images of the surface of epidermal cells of *Nicotiana benthamiana* transiently co-expressing full-length REM1.2 (magenta) and/or REM6.1 (green) versus truncated REM1.2t (magenta) and/or REM6.1t (green). Scale bar = 4 μm. Co-exclusion of AtREM1.2/AtREM6.1 and AtREM1.2t/AtREM6.1. Truncated versions of the remorins correspond to AtREM1.2_118-213_ and AtREM6.1_347-487_. Pearson’s correlation coefficient was calculated from at least 23 cells over the course of three independent experiments. Significant differences were determined using a Kruskal–Wallis test followed by a Dunn’s multiple comparison test. Different letters indicate significant differences (*p* > 0.01). **b** Confocal images of *N. benthamiana* epidermal cells expressing GFP-StREM1.3 (secant view) and GFP-StREM1.3___EEE (Z projection) with mutations at positions L126E, L137E, and L155E. **c** The upper two panels show RhodPE-labeled GUVs (magenta) with bound GFP-StREM1.3_86-198_ (green), and the lower two panels show the absence of binding of GFP-StREM1.3_86-198__EEE. **d** Structural ensembles (5 structures) of AF2 dimer predictions for the C-terminal region of remorin family members. Structures are colored in pLDDT values, and the code is indicated aside. **e** Surface electrostatic potential of a selected dimer the ensemble shown in d. **f** Two examples of multimer predictions for >4 monomers (supplementary multimers are shown in Supplementary Fig. 9a). Coloring is analogous to (**d**).

In the hypothesis that the C-terminal coiled-coil domain and the N-terminal IDR may resume dedicated roles during membrane association and nanodomain clustering, we then used dual-color TIRF on *Nicotiana benthamiana* leaves expressing a truncated version of AtREM1.2 (AtREM1.2t, AtREM_118-213_) and/or AtREM6.1 (AtREM6.1t, AtREM_347-487_), containing the C-terminal regions including the membrane anchor and the oligomerization domain, lacking the N-terminal IDR based on the structure predictions by AF2 (M&M subsection: Cloning, Plant culture and protein expression, Dual-color TIRF microscopy, Statistics and reproducibility). The truncated AtREM1.2t and AtREM6.1t remain localized in distinct nanodomains. However, interestingly, the fluorescence pattern observed in the truncated REM pair showed a different AtREM1.2t/AtREM6.1t distribution. AtREM6.1 clustering increases when truncated and, as they co-exclude, AtREM1.2 is showing the opposite trend (Fig. 4a and Supplementary Fig. 7). A comparable pattern is observed, when the truncated version AtREM1.2t and the intact AtREM6.1 are co-expressed, the proteins still segregate in distinct nanodomains and the nanodomain clusters of AtREM6.1 appears denser. However, the effect of increased clustering is less pronounced than in the case of AtREM1.2t/AtREM6.1t co-expression (Fig. 4a and Supplementary Fig. 7). Our data suggest that (1) the C-terminal region alone, containing the coiled-coil and the REM-CA domains, is sufficient for REM nanodomain exclusion and (2) the N-terminal domain can modulate nanodomain clustering.

### Structures of a C-terminal coiled-coil domain

We previously observed the occurrence of oligomeric assemblies in REM constructs of StREM1.3, including the predicted coiled-coil region^5,7,8^, and further that the formation of assemblies has a significant impact on REM membrane association^7,9^. This observation was confirmed for other REM groups and REMs of different organisms^6,21^. Indeed, using confocal microscopy, we observe that a triple mutation L/E at hydrophobic positions in the coiled-coil region of StREM1.3_86-198_ (L126E, L137E, and L155E) impairs membrane association and nanodomain clustering in vivo on *N. benthamiana* leaves (Fig. 4b) and in vitro on membrane-mimicking giant unilamellar vesicles (GUVs) (Fig. 4c)^7,9^ (M&M subsection: Protein production and purification, Giant unilamellar vesicle (GUV) preparation, fluorescence microscopy on GUVs).

Considering the predominant role of the C-terminal region for membrane association and nanodomain co-exclusion, we searched if multimerization over a coiled-coil motif reflects a conserved feature of the C-terminal region within the REM family. We performed AF2 multimer predictions, including selected sequence segments of the coiled-coil region, its extensions, and the REM-CA for REMs of the different groups, based on the prediction of the helical region by AF2 (Supplementary Fig. 2) (M&M subsection: Sequence alignment and structure prediction). Indifferent to the considered construct and the number of initial monomers (2 ≦ monomer number ≦ 4), AF2 predicts antiparallel coiled-coil dimer complexes with a high pLDDT confidence score for REMs shown for the structural ensembles of five structures in Fig. 4d. We note that, in some cases, the predictions yield a structure that does not align with the structural ensemble and represents low pLDDT values (e.g., AtREM6.4). The dimers exhibit a slight left-handed pitch, but do not reflect the pitch angle resulting from the classical heptad repeat of hydrophobic residues^33^. The repeat (HPPHPPP)_n_ with a seven-residue consensus repeat of hydrophobic (H) and polar (P) residues, is not observed in REM sequences (Fig. 1b and Supplementary Fig. 1), but a repetition of hydrophobic vs. polar residues still characterizes the segments including predicted coiled-coil motifs. The knob-into-hole hallmark of coiled-coil structures^33–35^ with hydrophobic amino acids intercalating into a hole in the interfacing α-helical structure applies to the coiled-coil zipper in the REM family members of all groups, as exemplified for StREM1.3 in Supplementary Fig. 8a. AF2 dimer prediction of the triple mutant StREM1.3_86-198 EEE_ or StREM1.3_86-198 PPP_ at positions L126, L137, and L155 still yield an antiparallel coiled-coil dimer with a lower pLDDT confidence score (Supplementary Fig. 8b). Interestingly, the coiled-coil dimers of all family members exhibit a specific curvature.

The surfaces of the dimeric coiled-coils are selectively charged, with a positive and a negatively charged surface reminiscent of a barcode (Fig. 4e). The positive charges are concentrated on the dimer surface representing a positive curvature.

Because REM constructs, lacking the IDR, can form supra-molecular filamentous assemblies^6,7,21^, we tested AF2 predictions for multimer assembly of REMs, including the coiled-coil domain and C-terminal anchor in multiple configurations. AF2 predicts an antiparallel/parallel coiled-coil trimer for shorter fragments (amino acid number ≦20) of the StREM1.3 coiled-coil domain with a low confidence pLDDT score when processing the multimer prediction of three monomers. When increasing the number of monomeric structures, including the entire coiled-coil domain (StREM1.3_67-198_), AF2 predicts different kinds of assemblies with low confidence score (Fig. 4f and Supplementary Fig. 9). One parameter that remains constant for the higher-order multimeric assemblies (>16), which we tested, seems the arrangement of monomeric subunits parallel to the filamentous axis, organizing the intercalating monomer interface to create curvature along the axis. These results are underpinned by the predictions of trimeric or tetrameric coiled-coil propensity for REMs by the recently developed prediction server Coconat^36^ (Supplementary Data 3). They indicate an adaptability of REMs towards different states of the coiled-coil oligomerization depending on the local molecular environment. Consistently, REM constructs containing mutations that target the coiled-coil knobs in StREM1.3 are impaired in membrane association in vitro and in vivo (Fig. 4b, c)^7,9^.

Taken together, our data suggest that REMs of all six groups assemble into homodimeric coiled-coil structures, specific in curvature and charge distribution with a potential to assemble into higher-order filamentous structures. The N-terminal IDR is dispensable for controlling membrane association and co-excluding nanodomain localization, governed by the C-terminal coiled-coil assembly.

### Evolutional conserved pre-structuration and dynamics of the remorin dimers

**Fig. 5:**
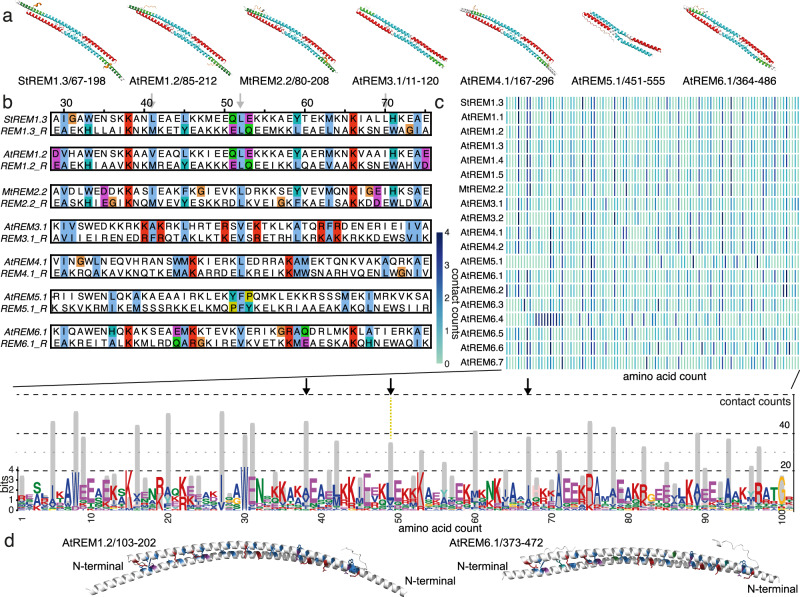
Conserved symmetric intermolecular coiled-coil contact motif. **a** Motif 1 (red) and 2 (blue) highlighted on AF2-predicted dimeric structures for selected REM C-terminal region (complete analysis shown in Supplementary Fig. 10). **b** Inverse alignment of the core region of the coil-coil domain centered on the symmetry axis (L137 in StREM1.3). **c** Atomic intermolecular contact counts per monomer in the dimeric assembly of each REM homolog (upper panel), cumulated over all REM homologs (lower panel, gray bars). Cumulative counts are highlighted on the conserved amino acid pattern of motifs 1 and 2. **d** Residues with ≧35 cumulative contact counts are shown in stick representation on AtREM1.2 and AtREM6.1 (color coding as in 5b).

Since the structures predicted for the coiled-coil dimers of all remorins resemble all the family members considered in this study (Fig. 4d), we were intrigued about the conserved motif distribution within the coiled-coil region. The recurring structural pattern of motifs 1 and 2, centered on the predicted coiled-coil domain (Fig. 5a and Supplementary Fig. 10), suggests a conserved dimerization motif. We therefore performed an inverse alignment of the REM sequences by selecting the confronting residues at the endpoint of the dimeric coiled-coil domain (Fig. 5b and full representation Supplementary Fig. 11). We observe the conserved location of the coiled-coil region exhibiting a symmetric distribution centered around the selected conserved hydrophobic residue situated in motif 1 (Fig. 5b and Supplementary Fig. 1), marking the zipping center of the antiparallel coiled-coil motif. This showed that the symmetry axis of the coiled-coil zipper can be localized to a hydrophobic residue, conserved over the REM family members. This residue corresponds to StREM1.3 L137, which we observed to have an important impact on membrane association. Because prediction results indicate that REMs might be capable of adopting different structural configurations (Supplementary Data 3), we searched whether there is a conserved symmetry in the inverse alignment. Several of the hydrophobic interface residues are arranged in a symmetric distribution, indicating that parallel or antiparallel configurations of these residue stretches appear plausible (Fig. 5b).

We used the AF2-predicted structures to search for a conserved motif of intermolecular interactions. We quantified the intermolecular atomic contacts <3 Å for all REM AF2 structures (Fig. 5c, upper panel), revealing similar as well as distinct intermolecular contact patterns (M&M subsection: Contact Map Analysis). To distinguish the common intermolecular contact motif, we then counted the total contacts observed in the group members for each aligned residue and compared them to the conserved motif pattern (Fig. 5c, lower panel). We found the symmetric motif to be composed as follows, HX_40/41_HX_18/19_HX_11_H_C_HX_11_HX_18/19_HX_40/41_ with H, X, and H_C_ standing for hydrophobic residue, unspecific amino acid and the central hydrophobic residue, respectively. The major sites with contact counts >35 are displayed on the AtREM1.2 and AtREM6.1 C-terminal CC structures which do not co-localize in vivo, depending solely on the C-terminal region (Fig. 4a).

**Fig. 6:**
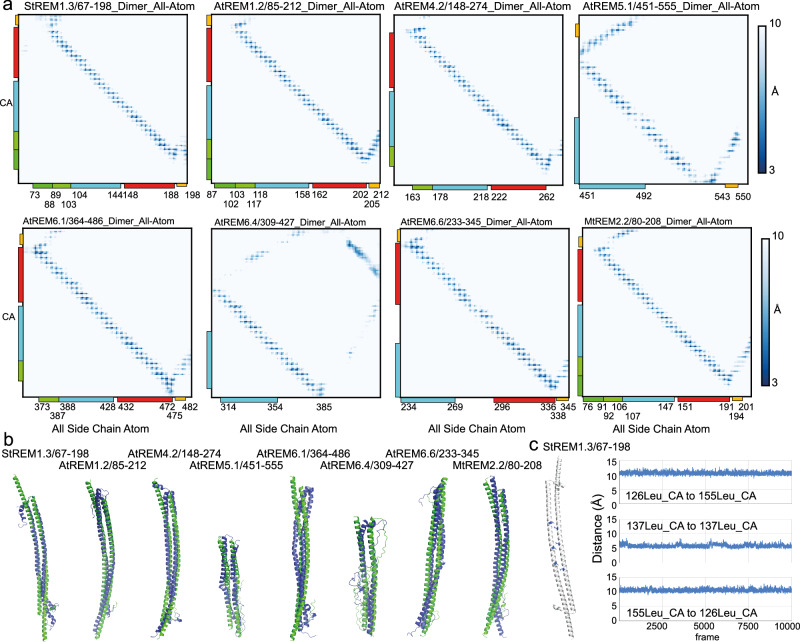
Robustness of the coiled-coil contact motif. **a** Intermolecular contact map of Cα of monomer 1 and all atoms of monomer 2 (range 3–10 Å) of the structures adopted throughout the 1 μs atomistic MD simulation based on the Amber forcefield^38^, indicated aside are the conserved Motifs 1 and 2 (residue-specific molecular contacts detailed in Supplementary Fig. 12). The blue scale is decoded in the right-hand panel. **b** AF2-predicted dimeric coiled-coil structures at the beginning (green) and at the end (blue) of 1 μs atomistic MD simulation. **c** Distance plot of the Cα-Cα contacts over 1 μs atomistic MD simulation of three selected residues of StREM_67-198_ (right panel), highlighted on the structure in the left panel (Leu126_monomer_1_-Leu155_monomer_2_, Leu137_monomer_1_-Leu137_monomer_2_, and Leu155_monomer_1_-Leu126_monomer_2_).

To test the stability and the dynamics in the CC dimerization domain, and their conservation, we turned to atomistic MD simulations^37^ based on the AMBER ff14sb forcefield^38^, using the dimeric CC structure predicted by AF2. We chose the Amber ff14sb force field and classical MD simulations because this is computationally less demanding (M&M subsection: Molecular dynamics simulation using Amber fields). We performed simulations in an aqueous solution over 1 μs on the structure of the C-terminal region (CC plus C-terminal anchor) for the REM with the, to our knowledge, best-studied structural features, StREM1.3_67-198_. We observe a very stable antiparallel dimer complex remaining in the dimeric configuration and maintaining the bending tendency, which was observed on the AF2-predicted structure, over the simulation (Fig. 6a).

We then tested dimer stability of selected dimers of the REM groups using atomistic MD simulation over 500 ns, namely StREM_67-198_, AtREM_85-212_, AtREM4.2_148-274_, AtREM5.1_451-555_, AtREM6.1_364-486_, AtREM6.6_233-345_, AtREM6.4_309-427_, and MtREM_80-208_.

The stability of the coiled-coil dimers of all family members is conserved over the simulation period as reflected by similar structures at the beginning and at the end of the simulation run (Fig. 6b). We quantified interatomic contacts at the dimer interface by monitoring the minimal distances between 3 and 10 Å adopted throughout the 1 μs simulation^39^. About 3 Å was selected as the lower intermolecular atomic distance limit because we could not map any distance below. The distribution profile of intermolecular atomic contacts is rather uniform over the coiled-coil domains of all REM family members, shown for CA-C (all carbons) contacts in Fig. 6b (for a detailed view, see Supplementary Fig. 12), revealing the intermolecular contacts in a helical configuration. To compare the performance of AMBER ff14sb with AMOEBA for simulation of the long CC domain, we performed simulations of the CC dimer using AMOEBA (Supplementary Fig. 13, M & M subsection: Molecular dynamics simulation using the polarizable force field AMOEBA and Adaptive sampling) for the StREM_67-198_. The results are very similar for both force fields, underscoring the stability of the CC structuration and potentially reflecting only a few differences between the forcefields due to the strong contribution of hydrophobic interaction to the structural motif. Some differences are observed within the REM-CA region which is consistent with the higher flexibility and an increased contribution of polar residues to the structuration of this region. The contacts established during the simulation indicate the stability of the initial structure, which shows a similar interatomic contact distribution reflecting the helix conformation, as detected by MAPIYA^40^ (Supplementary Fig. 14).

Our next goal was to map and compare the dynamics in the coiled-coil region of the REM family members. We selected the three conserved hydrophobic residues in the coiled-coil domain because we had confirmed their impact on membrane association of the C-terminal region of StREM1.3 and measured the fluctuation of distances over the simulation run. The distances between the selected hydrophobic residues, Leu126-Leu155, Leu137-Leu137, and Leu155-Leu126 in StREM1.3, fluctuate slightly, maintaining the intermolecular atomic contacts relatively stable (Fig. 6c).

The distances of the respective aligned hydrophobic residues in the coiled-coil domains of the other REM family members show similar relatively constant profiles with an exception for the central Leu490-Leu490 contact in AtREM5.1, exhibiting a lower AF2 confidence in this segment and a higher MD distance fluctuation (Supplementary Fig. 15).

### Tunable fuzzy coats decorate remorin dimers

**Fig. 7:**
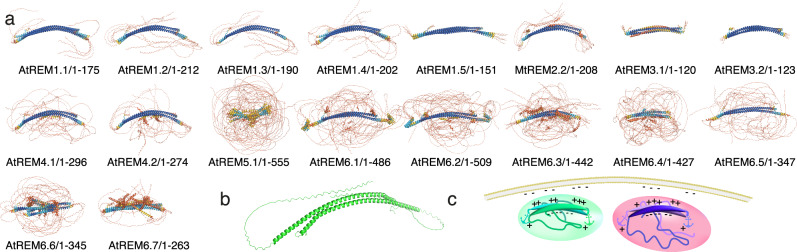
Full-length remorin pre-structuration. **a** Structural ensembles (five structures) of AF2 dimer predictions for REM family members. Structures are colored dependent on the pLDDT score (blue: pLDDT >90; cyan 90 > pLDDT > 70; yellow 70 > pLDDT > 50; red pLDDT < 50 values as in Fig. 4d). Supplementary Fig. 16 shows structures of each monomer colored by pLDDT and conserved sequence motifs 1 and 2. **b** Model of StREM1.3 architecture, constructed by artificially combining the NMR structure of StREM1.3_150–198_ with the predicted AF2 dimer structure StREM1.3_1–198_ truncated at residue 150. **c** Cartoon model of how two members of evolutionary distant REM groups approach distinct membrane spots enriched in anionic lipids such as PIPs.

When REMs associate with the membrane, containing specific anionic lipids such as PIPs capable of segregation^41,42^, REMs must be available to establish the necessary electrostatic and hydrophobic interactions with the membrane surface. All members of the REM family contain an N-terminal IDR of variable length, including up to 400–450 amino acids (Fig. 1d and Supplementary Fig. 2). We performed AF2 multimer predictions of the full-length REMs to assess how the pre-structuration of the IDR might impact on the structural arrangement of the coiled-coil domain and the full-length protein when comparing the different members of the REM family. Full-length REM homologs were exclusively predicted as dimers by AF2, containing the conserved coiled-coil α-helical dimerization motif (Fig. 7a), such as for the C-terminal region in isolation. The location of the dimerizing motifs is conserved (Supplementary Fig. 16). For all homologs, the pLDDT score within the coiled-coil domain is higher in the absence of the IDR (Supplementary Fig. 17).

In the case of an IDR, pre-structuration would imply only the definition of a sampling space and potential motif formation of the conformational ensemble adopted by the IDR. It is worth noting that Alphafold (2021) had a tendency to overestimate the radius of gyration of the conformational space as assessed by SAXS measurements^43^. AF2 multimer predictions indicate coiled-coil dimer assembly, with the IDR sampling a confined space around the C-terminal region (coiled-coil domain and REM-CA) and sampling structural motifs for several family members (Figs. 7a, 1b). Although the IDR as such does not necessarily lead to a chain compaction occurring in only 2% of >20,000 disordered chains^44^, the coiled-coil assembly restrains the conformational space accessible to the IDR.

The C-terminal region is, therefore, surrounded by a tunable electrostatic envelope reminiscent of the fuzzy coat observed for amyloid fibrils^45–48^. A polyelectrolyte fuzzy coat can control cellular mechanisms and functions, such as interactions with the membrane or partner proteins dependent on the environmental conditions (pH, salt, etc.)^45–48^. It can promote and modulate physico-chemical phenomena, including liquid-liquid phase separation, which REMs could trigger on the membrane surface^9^. REMs contain numerous phosphorylation sites in the IDR^28^, providing an additional source to control the multivalent interactions of fuzzy coat, coiled-coil domain, and interactions with molecular partners.

While the predictors Multicoil2^26^ and AF2 agreed approximately on the coiled-coil region of REM homologs, in a dimeric configuration in the case of AF2, the recently reported Coconat^36^ algorithm predicted a rather variable set of coiled-coil architectures, including antiparallel dimers, trimers, and tetramers or the absence of coiled-coil assembly (Supplementary Data 3). We performed the Coconat^36^ prediction only for the full-length REMs without testing different constructs, which might impact the prediction results.

Because AF2 suggested the preservation of a dimeric structure in full-length REMs in an environment without any molecular partners, we used Dali^49^ to test the structural homology for the different REM C-terminal dimers, lacking the IDR. Because function is usually related to the underlying structure, structural homology should provide ideas about functional or mechanistic implications. As presented for the best matching 10 structures of the restrained PDB25^50^ set for AtREM1.2, AtREM6.1 and StREM1.3 in Supplementary Fig. 18 and reflected by the significant number of structural homologs in this dataset and in the full PDB (625 and 1288, respectively for StREM1.3, Supplementary Data 2), the structural motif of the coiled-coil dimer is represented in numerous biological complexes. Several structural homologs of the C-terminal region possess the capacity to participate in multiple coiled-coil configurations or dynamic mechanistic processes, such as synaptobrevin, prohibitin, or myosin (Supplementary Fig. 18).

Taken together, our data support a conserved REM model, in which the coiled-coil domain and the REM-CA adopt a pre-structured dimeric architecture. This CC domain is surrounded by a fuzzy coat in the absence of molecular partners, such as it is shown for the StREM1.3 model, constructed from the NMR structure of StREM_150-198_ and the AF2 dimer prediction (Fig. 7b). The coiled-coil domain and REM-CA pre-structure with varying dynamics between REM family members, including one kink region centered on a conserved Gly residues and a second slight kink with increased flexibility in the region of a partially conserved Gly. The electrostatic barcode of positive charges and the IDR fuzzy coat provide a tunable selective entity that could guide the arrangement of a specific environment when segregating membrane nanodomains.

## Discussion

REMs of group 1–6 sustain a wide range of cellular functions^11^, segregating into liquid-ordered nanodomains^9,10,15^, which are enriched in specific anionic lipids and sterol on the surface of the plasma membrane, and are modulated by the lipid composition and the protein phosphorylation status^9–11,15^. To do so, multimerization^7,21^ and anchoring by a C-terminal membrane anchor REM-CA^15^ to specific anionic lipids, such as PI4P for StREM1.3, and acylation REM-CA for several members play essential roles^19,20^. Acylation impacts on membrane association of REMs^15,19,20^, while nanodomain segregation is independent^19^. Choosing their specific molecular partners during membrane association REMs creates potent signaling hubs for selected signaling pathways^13,14^. Evidence for StREM1.3 supports the hypothesis that REMs target their functional site on the plasma membrane independent of secretory pathways^15^. REMs might thus rely on a structural arrangement in the cytosol before associating to a membrane surface to guide each REM to its specific cellular localization^15^. We here propose a conserved pre-structuration mechanism REMs to target nanodomain organization, guided by two conserved coiled-coil dimerization motifs and a pre-structured C-terminal anchor, and regulated by the IDR before encountering the membrane surface.

Hypothesizing that the REM architecture is responsible for their selective co-localization or mutual exclusion in nanodomains^13,14^, we first assess the architecture of monomeric REM family members of the groups 1–6 and show their common sequence motif as well as monomeric structure motif distribution. REM-CAs contain a common sequence motif, independent of the acylation sites suggesting a common pre-structuration. We determined the NMR structures of selected REM-CA peptides of groups 1–6, containing the conserved REM-CA motif. Our data suggest a common underlying structure containing a kink at a conserved Gly at the C-terminal ending. A second slight kink at a Gly in StREM1.3, located in the N-terminal sequence adjacent to REM-CA, indicates a relatively conserved second flexibility spot and potential kink in several groups of the REM family. Atomistic molecular dynamics simulation confirms the robustness of the preformed REM-CA structures, further highlighting different dynamics between the family members, likewise reflected in distinct RMSD of the NMR structures. Our MD simulation data indicate that the polarizable force field yields the most coherent data when compared to the NMR data on REM-CAs. This is consistent with the purpose of polarizable forcefields to more accurately represent polar contributions^32^ and the observation of REM-CA peptides to present the more flexible structures, to include polar residues in the structured motifs, and to adapt its structure for subsequent interactions with the membrane surface relying on electrostatic interactions^1^.

Pre-structuring of the REM-CA could facilitate its recognition of the specific lipid headgroups^7,10,15,21^ and their efficient interactions to anchor the REMs despite the required energetically unfavorable re-arrangement of the IDR towards a restrained space at the surface of the membrane. Whether one or multiple Cys, present in a subgroup of REM-CA, are S-acylated, can significantly increase and tune membrane affinity without primarily affecting the molecular segregation of selected lipids^19,20^.

We could establish that the C-terminal region alone, lacking the IDR, is responsible for co-localization and mutual exclusion of nanodomains and efficient membrane association in vivo and in vitro for REMs from distant groups. Among all REM groups, the C-terminal region of REMs assembles into antiparallel coiled-coil dimers with a high confidence score as predicted by AF2. The previously predicted coiled-coil region in StREM1.3^8^ overlaps with the segments identified in this manuscript and is part of the dimerization motif predicted by AF2.

The coiled-coil dimers exhibit curvature and selective charge distribution with one predominantly positive and one predominantly negative charged surface. The positive charges are principally located on the positively curved dimer surface, reminiscent of a charge barcode. The charge distribution of the positively charged dimer surface resembles a barcode pattern, specific for each REM. There are no obvious similarities in the barcode patterns between the members of a specific REM group. The barcodes could contribute to the preferential association to specific lipid headgroups, such as we have shown for StREM1.3, which requires PIPs, notably PI4P, or phosphatic acid (PA) to associate to the membrane as well as β-sitosterol to cluster into nanodomains in vitro and in vivo^9,15^. Furthermore, differences in barcode patterns could promote the selective preferences to target different nanodomains during membrane association, impacting the co-localization behavior.

Distinct individual intermolecular atomic contacts mapped over the primary sequence could further explain the exclusive nanodomain distribution, whereas the conservation of REM dimerization relies on a conserved symmetric dimerization motif HX_40/41_HX_18/19_HX_11_H_C_HX_11_HX_18/19_HX_40/41_ containing a hydrophobic at the center H_c_. Atomistic MD simulations further underpin the arrangement of the C-terminal region over the conserved coiled-coil dimerization motif to reflect a robust assembly in the absence of molecular partners with relatively homogeneous dynamics when comparing different REM groups.

The N-terminally located relatively conserved flexibility spot in REM-CA towards the adjacent coiled-coil zipper provides liberty to the zipper to re-arrange the antiparallel coiled-coils containing the N-terminal IDR to minimize the energy cost upon membrane contact. The conservation of the symmetry of major hydrophobic contacts could represent an additional molecular asset to re-arrange the C-terminal coiled-coil domain. This hypothetic liberty towards different intermolecular arrangements on the membrane surface is further supported by prediction results that indicate distinct configurations for REM family members. The capacity of the C-terminal region to adopt multiple multimeric assemblies with a low confidence score predicted by AF2, which have been observed in vitro^6,7,21^, is in favor of a structural plasticity of the coiled-coil domain. When encountering the membrane surface, the C-terminal region of REMs could anchor REM-CA and re-adapt its configuration towards energetically more favorable states. While AF2 predictions, using a clustered subset of aligned sequences, can assess different structural states^51^, the here-considered second structural state would occur in contact with the membrane and a specific lipid partner and should not be accessible to AF2 predictions. The search for structural homologs of the C-terminal region further supports the adaptability of the hydrophobic coiled-coil zipper when encountering partner molecules. Adapting the structural arrangement is generally a prominent feature of coiled-coil zippers, such as Myosin II motor proteins^52^ or SNARE complex subunits^53^, and characteristic of several homologs of the REM C-terminal region.

REMs contain a N-terminal IDR of significant variable length (up to approx. 450 amino acids), source of their classification into six groups^5^, suggesting an impact of the IDR on the assembly and pre-structuration of REMs. The REM IDR coordinates REM regulation, mediated by protein–protein interactions^54^ and phosphorylation events^11,28^.

Our results indicate that the conserved dimeric coiled-coil motifs assemble in the presence of the IDR, independent of its size and, thereby, of the REM group membership. However, the presence of the IDR engenders a lower confidence score in the predicted structures as compared to the C-terminal region, consistent with the IDR’s regulatory role. Consistently, the IDR does not impact on effective REM nanodomain co-localization or exclusion, but modifies the observed nanodomain aspect and confers more efficient exclusivity during nanodomain segregation.

Two major conserved sequence motifs occur in the IDR, predicted as α-helical with a low confidence score by AF2, suggesting a conserved spot of transient or conditional structuration^55^. AF2 predictions of IDRs can be related to the compaction of the IDR when considered in isolation^44^, while here, the IDR is covalently linked to a dimeric coiled-coil. However, the IDR compactness and the differences between REM family members might still be reflected in the predicted IDR ensembles presented here. The IDR presents an adjustable polyelectrolyte fuzzy coat around the REM coiled-coil domains in the absence of a molecular partner, reminiscent of the fuzzy coats observed in amyloid assemblies^45–48^. IDRs are highly represented in molecular entities capable of promoting liquid-liquid phase separation^56^. REM-driven condensate formation might not only occur on the surface of the membrane^9^ but could indeed be a cellular feature of certain members of the protein family.

Our study reports a pre-structuration of dimeric remorins before establishing contact with the membrane, based on modeling, solution NMR spectroscopy and atomistic MD simulations. This opens an avenue to study the structures of remorins on the membrane surface by solid-state NMR and to analyze the remorin-membrane interactions using coarse grain MD simulations, contributing to the understanding of the functional mechanisms in vivo.

Taken together, our data indicate that the pre-structuration in the cytosol of remorins of all six family groups into homodimeric antiparallel coiled-coil assemblies containing a pre-folded C-terminal anchor, is a pre-requisite to associate to the membrane lipid headgroups and cluster into membrane nanodomains. The surface charge distribution of the coiled-coil domain and the electrolyte fuzzy coat in membrane-targeting REMs could facilitate and tune the association of the C-terminal anchor to lipid headgroups and the membrane, respectively.

This ensemble of candidates relying on the REM structure composition represents a powerful set of devices for the cell, highly sensitive to local cellular regulation such as pH or salt and to post-translational modifications or interactions with a molecular partner.

## Materials and methods

### Cloning

REM1.2 and REM6.1 sequences were previously published^14,28^. REM1.2_118-212_ and REM6.1_347-486_ were generated by site-directed mutagenesis using REM1.2 or REM6.1 as templates, respectively. The following primers were used:

REM1.2_118-212__Fw: GGGGACAGCTTTCTTGTACAAAGTGGCCGCAGAGAACAAAGCTGAGAA

REM1.2_118-212__Rv: GGGGACAACTTTGTATAATAAAGTTGGTTAGAAACATCCACAAGTTGCCTTT

REM6.1_347-486__Fw: GGGGACAGCTTTCTTGTACAAAGTGGCCGCTAGCAAGGAGGATGAAGA

REM6.1_347-486__Rv: GGGGACAACTTTGTATAATAAAGTTGGTTAAGAACAAAAGCTAAAGCAAGAG

Full-length and truncated REM1.2 and REM6.1 sequences were cloned into pDONR P2RP3 using Gateway BP reaction (www.lifetechnologies.com). Multisite Gateway cloning was used to clone full-length or truncated REM1.2 or REM6.1 with pUbi10 and either the mRFP1.2 tag or the mVenus tag, using pLOK180_ pR7m34g as the destination vector.

All constructs were propagated using the NEB10 E. coli strain (New England Biolabs).

### Plant culture and protein expression

*Nicotiana benthamiana* plants were cultivated in a greenhouse at 25 °C with a 16-h photoperiod. For transient expression, *Agrobacterium tumefaciens* (strain GV3101) carrying the tested constructs were cultured at 28 °C overnight on a selective medium. Agrobacteria were infiltrated in the leaves of 3-week-old plants, and plants were observed 48 h after infiltration.

### Dual-color TIRF microscopy

For quantification of TIRF microscopy images, three independent experiments were conducted. The observation of plants transiently expressing constructs that were infiltrated at the same moment is considered an independent experiment. Within an independent experiment, at least seven cells are observed per condition.

Forty-eight hours after infiltration, a small sample of the leaf was mounted between a slide and cover slip with a drop of water to maintain tissue hydration. Image acquisitions were done on an inverted motorized microscope Nikon Ti Eclipse equipped with a 100 Å oil-immersion PL-APO objective (NA = 1.49), a TIRF arm and a sCMOS Camera FUsion BT (Hamamatsu). 488 nm and 561 nm lasers were used to image mVenus- and mRFP1.2-tagged constructs, respectively. The laser angle was adjusted for each laser to obtain the highest signal-to-noise ratio. Image analysis was performed with Fiji using the Coloc2 plug-in. Selection of a region of interest as well as background subtraction was done to limit background noise, then co-localization analysis was done using Costes threshold regression, with 10 Costes randomizations.

At least 23 cells were analyzed for each condition over the course of three to four experiments.

### Statistics and reproducibility

Data were plotted using R (cran.r-project.org). Kruskal–Wallis followed by Dunn’s multiple comparison test was performed using Prism 6.0 (GraphPad). *p* values are given for the displayed graphs in the respective Figure legends and further details on the experiment replications in the previous section.

### Sequence alignment and structure prediction

Sequence alignment and visualizing was performed by Blast (NCBI) and Clustal Omega^57,58^.

For the structural prediction of our protein of interest, we employed ColabFold v1.5.2-patch^24^, an implementation of AlphaFold2^23^ utilizing MMseqs2^59^ for sequence alignments, hosted on Google Collaboratory. The methodological approach facilitates the use of the structure prediction process for both monomers and complexes^24^. Solely the primary sequence information without structural templates was selected. “mmseqs2_uniref_env” was utilized as MSA Mode, leveraging the MMseqs2 database to generate multiple sequence alignments (MSA) capturing environmental sequence diversity. The pair mode was selelected “unpaired_paired” accommodating unpaired and paired sequence information in the prediction process to enhance complex structure prediction accuracy. The model type was set to “auto” allowing the system to select the most appropriate model based on the input sequence. ‘alphafold2_ptm’ was used for monomer predictions, and “alphafold2_multimer_v3” for complex predictions, optimizing the prediction process for individual proteins and their complexes. The automatic number of recycles was chosen to refine the prediction. For “alphafold2_multimer_v3”, up to 20 recycles were permitted with a tolerance of 0.5. Post-prediction, the generated PDB formatted structures were meticulously sorted based on their average pLDDT^23^ scores, which measure the local accuracy of the predicted protein structures. This sorting allowed prioritizing structures with higher confidence levels in their conformational predictions. The choice of parameters was guided by the need to balance computational resources with the desire for reliable structural predictions.

### Coiled-coil prediction using multicoil2

Multicoil2 is a computational tool designed to accurately identify two- and three-stranded coiled-coil motifs within protein sequences^26^. The method relies on scoring matrices that consider the amino acid sequence’s propensity to form coiled-coil structures, providing a probabilistic output that indicates the likelihood and location of these motifs. The sequences listed in “remorin.txt” were used as input in Multicoil2. The analysis generated an output file detailing the coiled-coil possibility across the sequence. We reviewed this file to select intervals showing a relatively uniform and high probability of coiled-coil formation. This criterion was based on the continuity and consistency of the probability scores, which indicate a more substantial likelihood of coiled-coil regions.

### Motif prediction using MEME

Utilizing the MEME Suite version 5.5.2^22^, we identified motifs within the protein sequence provided in “remorin.txt”. We specified the search for seven motifs, aiming to comprehensively uncover significant patterns within the sequence without overwhelming the analysis. This number was chosen to balance thoroughness with analytical clarity.

#### Analysis and interpretation

MEME outputs a detailed report featuring seven identified motifs following the configured search. Each motif was evaluated for its sequence composition, statistical significance (e-value), and representation within the protein sequence. Motifs were correlated with their respective positions within the protein sequence, highlighting potential domains or sites of functional importance.

### Peptide synthesis and specification

The peptide samples utilized in this study were synthesized by GenScript. The peptides comprise the last 20 amino acids of ten selected remorin sequences and the extended C-terminal sequences (last 49, 39, and 28 amino acids) of StREM1.3. The details of the sequences are documented in “remorin_REM-CA.txt”. The peptides were synthesized to ensure high purity (≥80%).

### Peptide sample preparation for NMR

The samples were then dissolved in a solvent mixture of 90% H_2_O and 10% D_2_O at a concentration of 1 mM.

### NMR experiments and analysis

NMR experiments were conducted on an 18.8 Tesla (800 MHz ^1^H frequency) Bruker Avance Neo spectrometer. We utilized the 2D ^1^H-^15^N sofastHMQC^60^ experiment (pulse program: sfhmqcf3gpph, temperature: 298 K, number of scans: 64), the 2D ^1^H-^1^H TOCSY (pulse program: dipsi2esgpph temperature: 298 K, number of scans: 8)^61,62^, to investigate the internal spin systems and their connectivity within peptides derived from remorin sequences. We utilized the Nuclear Overhauser Effect in ^1^H-^1^H NOESY Spectroscopy (pulse program: noesyesgpph, temperature: 298 K, number of scans: 64) to detect spatial proximities between hydrogen atoms. Spectra were referenced according to 4,4-dimethyl-4-silapentane-1-sulfonic acid (DSS) signal^63^. Spectra were processed using Topspin 4.1.

NMR resonance assignment was performed with CcpNMR Analysis 2.5.2^64^. The Chemical Shift Index (CSI) served to identify the secondary structure of the peptides. Chemical shift data were deposited at the BMRB under the following identifiers, StREM1.3_160-198 BMRB ID: 52390; StREM1.3_171-198 BMRB ID: 52391; StREM1.3_150-198 BMRB ID: 52393; AtREM1.1_156-175 BMRB ID: 52402; AtREM1.2_193-212 BMRB ID: 52403; AtREM1.3_171-190 BMRB ID: 52404; MtREM2.2_189-208 BMRB ID: 52405; AtREM4.1_277-296 BMRB ID: 52406; AtREM5.1_536-555 BMRB ID: 52407; AtREM6.2_490-509 BMRB ID: 52408; AtREM6.3_423-442 BMRB ID: 52409; AtREM6.4_408-427 BMRB ID: 52410; AtREM6.5_328-347 BMRB ID: 52411. To predict backbone dihedral angles φ and ψ, we employed the Dangle algorithm within CcpNMR. To generate the structural restraint list for structure calculation, we assigned the nuclear overhauser effects (NOEs). We used CcpNMR’s distance restraint generator, compiling lists of distance restraints.

### Structure calculation

The structure calculation was performed using CNS^29^, the process was initiated by integrating the distance restraint lists obtained from CcpNMR into the CNS environment. We prepared the input files for CNS by converting the distance and dihedral angle restraints into the format recognized by CNS. The initial models underwent a series of energy minimization steps in CNS to remove steric clashes and optimize the conformational energy. We employed simulated annealing protocols within CNS to refine the models further and explore the conformational space. This involves heating the system to a higher temperature and then gradually cooling it down, allowing the model to adopt energetically favorable conformations that agree with the experimental data. The CNS-based structure calculation process yielded a set of low-energy conformers representing the most probable three-dimensional structures of the peptides. Structures were refined using ARIA using water solvent during the refinement step^65^. The ensemble of calculated structures has been deposited for public access at the PDB for StREM1.3_171-198_ (PDB ID 9F1E), StREM1.3_160-198_ (PDB ID 9F1F), and StREM1.3_150-198_ (PDB ID 9F1G).

### Molecular dynamics simulation using Amber fields

#### System preparation

Our molecular dynamics (MD) simulations based on Amber ff99sb^66^ and Amber ff14sb^67^ force fields, in combination with the SPC/E water force field, were divided into two parts. The template structures for the monomer simulations were obtained by NMR structure calculation. For StREM1.3_171-198_ (PDB ID 9F1E), StREM1.3_160-198_ (PDB ID 9F1F), and StREM1.3_150-198_ (PDB ID 9F1G), a representative of the structural bundle deposited in the PDB was chosen. The template structures for the dimer simulations were obtained from AF2 multimer predictions. Each initial protein structure were solvated in a cubic box of explicit water molecules, ensuring a minimum distance of 2.0 nm between two images of the protein. Na+ and Cl- ions were added to a final concentration of 0.15 M. The details of all calculations performed are summarized in Table 2.

**Table 2:** Summary of the details of all calculations performed using Amber force fields

System type	Systems	System size	Total simulation time
Short	AtREM1.1 AtREM1.2 AtREM1.3 AtREM2.2 AtREM4.1 AtREM5.1 AtREM6.2 AtREM6.3 AtREM6.4 AtREM6.5	~10k particles	1 µs
Large	StREM1.3/171-198 StREM1.3/160-198 StREM1.3/150–198	~15k particles ~25k particles ~40k particles	1 µs
Dimer	AtREM1.2 AtREM4.2 AtREM5.1 AtREM6.1 AtREM6.4 AtREM6.6 MtREM2.2 StREM1.3	~1 M particles ~1 M particles ~450k particles ~700k particles ~550k particles ~1.4 M particles ~1.2 M particles ~1.2 M particles	1 µs

#### Molecular dynamics simulations

All simulations using non-polarizable force fields were performed using the GROMACS^68^ software, following a standard protocol to minimize, equilibrate, and execute the production run of the molecular system^69^. Energy minimization was performed using the steepest descent algorithm for 50,000 steps. Subsequently, the equilibrations under NVT and NPT conditions were performed at 300 K at 100 ps each, using a Van der Walls and Coulomb cutoff of 1.0 nm each. For equilibration and production runs, we used the velocity-rescaling thermostat coupled to the Parrinello-Rahman barostat (when relevant) with a time constant of 2.0 ps, a compressibility of 4.5 × 10^−5^ bar^−1^ and at a pressure of 1 bar. During the equilibration phase, the non-hydrogen protein atoms were restrained by a force constant of 1000 kJ mol^−1^ nm^−2^. Long-range electrostatics were modeled using the Particle-Mesh Ewald method. All bonds were treated using the LINCS algorithm. The integration timestep was 2 fs for equilibration and production.

#### Analysis

The secondary structures for the REM-CA peptides were calculated using the Timeline tool of the VMD software.

### Molecular dynamics simulation using the polarizable force field AMOEBA and adaptive sampling

System preparation for molecular dynamics simulation:

The initial structures were the same as the ones used for the AMBER simulations. For all systems, the residues have been protonated following the results of PROPKA3^70^. All systems were solvated in explicit water boxes using the xyzedit tool of the Tinker-hp distribution^71^, so that there was at least 30 Å between two images of the protein. The systems were then neutralized and NaCl atoms were added to reach 150 mM concentration. The details of all calculations performed are summarized in Table 3.

**Table 3:** Summary of the details of all calculations performed by AMOEBA force field and adaptive sampling

System type	Systems	System size	Total simulation time
Short	AtREM1.1 AtREM1.2 AtREM1.3 AtREM2.2 AtREM4.1 AtREM5.1 AtREM6.2 AtREM6.3 AtREM6.4 AtREM6.5	~35k particles	1.04 µs
Large	StREM1.3/171-198 StREM1.3/160-198 StREM1.3/150–198	~40k particles ~60k particles ~90k particles	1.04 µs
Dimer	StREM1.3	~460k particles	560 ns

#### Molecular dynamics simulations

In contrast to the M&M subsection “Molecular dynamics simulation using Amber fields”, based on force fields Amber ff14sb and Amber ff99sb^72^, we here used the polarizable force field AMOEBA^30^. All simulations were performed using the GPU version of the Tinker HP^71^.

Polarizable simulations were performed following a protocol previously published^30^. The force field parameters used for the protein parameters was the AMOEBA Polarizable force field for proteins^30^. Briefly, all molecular dynamics simulations were performed using the GPU version of the Tinker-HP software. During the calculations, periodic boundary conditions were employed using the Particle-Mesh Ewald method. The van der Waals and PME cutoffs were, respectively, of 12 and 7 Å. An analytical long-range correction of the vdW interactions has been used. The dipole convergence criterion of the preconditioned conjugate gradient polarization solver was set to 0.01 Debye/atom for the minimization steps, and to 0.00001 Debye/atom otherwise. For the minimization steps, no polarization or electrostatics terms were used. The systems underwent a minimization of 30,000 steps using an L-BFGS optimizer. The next equilibration steps were realized using a timestep of 1 fs, the RESPA integrator, and the Berendsen barostat (when relevant) unless stated otherwise. The solvent was then progressively heat up in the NVT ensemble, from 5 to 300 K using 10 K steps and spending 5 ps at each temperature, before undergoing an additional 100 ps at 300 K. The system was then allowed to slowly relax for three times 400 ps in the NPT ensemble while applying harmonic restraints of 10, 5, and finally 1 kcal/mol/A on the backbone atoms of the peptides. Then, all restraints were removed, and we used the Montecarlo barostat in combination with the BAOAB-RESPA1^73^ propagator. Four final equilibration steps were performed for 100, 200, 500, and 1000 ps by respectively increasing the outer timestep from 1 to 2 fs, 5 fs and finally 10 fs.

Regarding the production run, all calculations were performed in the NPT ensemble, using the Montecarlo barostat and the BAOAB-RESPA1 propagator with an outer timestep of 10 fs, and hydrogen mass repartitioning. The first 10 ns simulations was performed to generate the first set of structures. To maximize the phase space exploration, we then resorted to an adaptive sampling procedure: a number of structures were first extracted from this initial simulation to perform the first adaptive sampling round. The seeds were then chosen following a procedure already described in ref. ^74^. Briefly, a principal component analysis is performed on the 10 ns simulation using the scikit-learn^75^ and MDTraj^76^ packages from which the *n* = 4 first principal modes (over 10 calculated modes) are considered. The density ρ_k_ of the conformational space is then projected on the four modes and approximated using a Gaussian density kernel estimator:

$${\rho }_{k}\left({x}_{i}\right)=\frac{1}{{\left(2\pi {\sigma }^{2}\right)}^{n/2}{M}_{k}}{\sum }_{i=1}^{{M}_{k}}{e}^{-\frac{{\left|x-{x}_{i}\right|}^{2}}{2{\sigma }^{2}}}$$

With the σ bandwidth being chosen with the D.W Scott method of Scipy^77^, M_K_ being the total number of configurations, x_i_ the orthogonal projection of the configuration on the n PCA modes.

Then a bias is introduced to the selection of a new seed x_i_ under the following form:

$$P(i)=\frac{{\rho }_{k}^{-1}\left({x}_{i}\right)}{{\sum }_{j=1}^{{M}_{k}}{\rho }_{k}^{-1}\left({x}_{j}\right)}$$

The probability of selecting the x_i_ structure is inversely proportional to its density, projected on the first four PCA components, favoring new, undiscovered structural states.

Following this, 10 ns simulations were run to form the new phase space of structures for the next adaptive sampling round. For each following round, all simulations are added to the conformational space on which the next adaptive sampling are performed. The number of seeds used for each round is summed up in the Table 4.

**Table 4:** Number of seeds used for each round of simulation

Systems	1st	2nd to 7th	Total simulation time
Short	8 × 10 ns	16 × 10 ns (7 rounds in total)	1.04 µs
Large	8 × 10 ns	16 ×10 ns (7 rounds in total)	1.04 µs
Dimer	8 × 10 ns	16 × 10 ns (4 rounds in total)	560 ns

#### Note regarding the dimer simulation

The StREM1.3 Dimer system represents one of the largest systems to be simulated so far using the AMOEBA force field. If solvated in a cubic box, the number of particles in the system would approximately reach 1 million atoms. To reduce this number, we restrained in space the ten first residues of one of the two monomers by using a positional restrain of 10 kcal/mol/A. This prevented the dimer from rotating, allowing us to use a rectangular box, and thus reduce the number of particles to c.a.460k. The other parameters of the simulation were otherwise identical to those of the previous systems.

#### Analysis

The secondary structures for REM-CA peptides were calculated using the Timeline tool of the VMD software. Regarding the polarizable simulations, the observables had to be reweighted to take into account the bias introduced by the adaptive sampling procedure. For this purpose, the unbiasing factor α_i_ of each seed is defined as:

$${\alpha }_{i}=\frac{1}{{M}_{k}P(i)}$$

The final weight of each seed is then normalized:

$${\omega }_{i}=\frac{{\alpha }_{i}}{{\sum}_{j} \, {\alpha }_{j}}$$

### Contact map analysis

To compare the structural stability of the coiled-coil domains, contact maps were generated based on the alpha carbons to all side chain carbon (Cα and all side chain carbon) distance between helices, using a cutoff of 3 Å.

### Protein production and purification

GFP-REM1.3_86-198_ and GFP-REM1.3_86-198__EEE were constructed as follows from N- to C-terminal: 6His-GFP^78^-linker(LESTSPWKKAGS)-REM1.3_86-198_ (wild-type or L125E/L137E/L155E EEE). The corresponding DNA sequences were ordered from Eurofins Genomics (Louisville, KY, USA) and cloned in pET-24a between NdeI and XhoI. They were produced in BL21-DE3-pLys cells in a lysogeny broth medium with 30 μg/mL kanamycin. At OD600 = 0.6, 1 mM IPTG was used to induce protein expression at 18°C overnight. Cells were pelleted at 6000×*g* for 20 min at 4 °C, resuspended in lysis buffer (20 mM HEPES, 150 mM NaCl, 20 mM imidazole, 1 mM PMSF, and 0.02% NaN3 [pH 7.4] with complete protease inhibitor cocktail; Roche, Basel, Switzerland), and sonicated on ice. The lysate was centrifuged at 15,557× *g* for 30 min at 4 °C, and the supernatant was loaded on a HisTrap column (GE Healthcare, Chicago, IL, USA) equilibrated in 20 mM HEPES, 150 mM NaCl, 20 mM imidazole, and 0.02% NaN3 (pH 7.4). GFP-REM1.386-198 was eluted with elution buffer (20 mM HEPES, 150 mM NaCl, 400 mM imidazole, and 0.02% NaN3 [pH 7.4]) and dialyzed against 10 mM HEPES, 150 mM NaCl, and 0.02% NaN3 (pH 7.4) at 4 °C overnight, which triggered protein aggregation^7^. The turbid protein sample was centrifuged at 100,000×*g* for 2 h at 4 °C, the supernatant was discarded, and the pellet was resuspended in 10 mM HEPES, 10 mM NaCl, and 0.02% NaN3 (pH 7.4). After two more rounds of centrifugation, the last supernatant was kept and contained non-aggregated pure GFP-REM1.3_86-198_. Protein concentration was assessed by absorbance at 280 nm (Ɛ280 = 41,400 M^−1^ cm^−1^ according to Expasy ProtParam). All proteins were stored at 4 °C.

### Giant unilamellar vesicle (GUV) preparation

Lipids (50/26/8/16 mol % DPPC/DLPC/sitosterol/PIPmix) at 10 g/L were mixed in organic solvent with 1% (w/w) of di-oleoylrhodamine-phosphoatidylethanolamine (RhodPE). All lipids come from Avanti (Weston, FL, USA; dipalmitoyl-phosphoatidylcholine 850355, dilinoleoyl-phosphoatidylcholine 850385, sitosterol 700095), except for phosphoinositide mix (PIPmix) from bovine brain (P6023, Sigma-Aldrich, St. Louis, MO, USA). About 20 μL mixture were spread on Teflon disks, which were individually stored in small beakers. They were dried for at least 1 h under a vacuum with a desiccator. Using a bubbler, lipids were prehydrated under a stream of N2-saturated H_2_O for 20 min. About 5 mL 300 mM sucrose was gently layered on top of the disk (enough volume to cover it fully). From this point, care was taken not to shake the beaker to avoid breaking nascent GUVs. After overnight incubation at 34 °C, GUVs were collected using a severed pipette tip (to avoid shearing) and stored at 4 °C until further use. GUVs were stable for 1 week.

### Fluorescence microscopy on GUVs

Teflon-coated 50 μL observation chambers were coated with 5% BSA for 20 min at room temperature and then washed three times with 10 mM Tris and 150 mM NaCl (pH 7.4). Using a severed P200 pipette tip, a drop of the GUV suspension was deposited, followed by about 1.8.10–12 mol of GFP-tagged protein. A slightly elevated cover slide was installed using a double-tape face. Observations were carried out through optical oil on a Zeiss LSM 880 confocal laser scanning microscopy system (Leica, Wetzlar, Germany) equipped with argon, DPSS, and He-Ne lasers and a hybrid detector. GFP was excited at 488 nm, and RhodPE was excited at 565 nm.

### Reporting summary

Further information on research design is available in the Nature Portfolio Reporting Summary linked to this article.

## Supplementary information

Supplementary Information

Description of Additional Supplementary Materials

Supplementary Data 1

Supplementary Data 2

Supplementary Data 3

Supplementary Data 4

Supplementary Videos

reporting summary

## Data Availability

Chemical shifts have been deposited under the following accession codes StREM13_160-198 BMRB ID: 52390; StREM13_171-198 BMRB ID: 52391; StREM13_150-198 BMRB ID: 52393; AtREM11_156-175 BMRB ID: 52402; AtREM12_193-212 BMRB ID: 52403; AtREM13_171-190 BMRB ID: 52404; MtREM22_189-208 BMRB ID: 52405; AtREM41_277-296 BMRB ID: 52406; AtREM51_536-555 BMRB ID: 52407; AtREM62_490-509 BMRB ID: 52408; AtREM63_423-442 BMRB ID: 52409; AtREM64_408-427 BMRB ID: 52410; AtREM65_328-347 BMRB ID: 52411. Structural ensembles have been deposited for StREM1.3171-198, StREM160-198, and StREM150-198 under the following identifiers PDB ID 9F1E, PDB ID 9F1F, and PDB ID 9F1G, respectively. Primary sequences of remorins used and detailed NMR restraints have been deposited in Supplementary data 1. Data used to create the Figures have been deposited in the Supplementary data 4. MD simulation data have been deposited in Zenodo under the following access: 10.5281/zenodo.14163696.

## Code availability

Software and code: CcpNMR Analysis 2.5.2 for NMR data analysis; CNS for structure calculation; Aria 2.0 for structure refinement; Multicoil2 for prediction of coiled-coil structure; Fiji using the Coloc2 plug-in; R (cran.r-project.org) Prism 6.0 for statistical analysis; Simulations were analysed using tcl code (available under 10.5281/zenodo.14163696) and the VMD software.

## References

[1] Gronnier, J. et al. Mechanisms governing subcompartmentalization of biological membranes. *Curr. Opin. Plant Biol.* **52**, 114–123 (2019). 31546133 10.1016/j.pbi.2019.08.003

[2] Reymond, P. et al. Cloning of a cDNA encoding a plasma membrane-associated, uronide binding phosphoprotein with physical properties similar to viral movement proteins. *Plant Cell* **8**, 2265–2276 (1996). 8989883 10.1105/tpc.8.12.2265 PMC161351

[3] Raffaele, S. et al. Remorin, a *Solanaceae* protein resident in membrane rafts and plasmodesmata, impairs potato virus X movement. *Plant Cell* **21**, 1541–1555 (2009). 19470590 10.1105/tpc.108.064279 PMC2700541

[4] Huang, D. et al. Salicylic acid-mediated plasmodesmal closure via Remorin-dependent lipid organization. *Proc. Natl Acad. Sci. USA* **116**, 21274–21284 (2019). 31575745 10.1073/pnas.1911892116 PMC6800329

[5] Raffaele, S., Mongrand, S., Gamas, P., Niebel, A. & Ott, T. Genome-wide annotation of remorins, a plant-specific protein family: evolutionary and functional perspectives. *Plant Physiol.* **145**, 593–600 (2007). 17984200 10.1104/pp.107.108639 PMC2048807

[6] Bariola, P. et al. Remorins form a novel family of coiled coil-forming oligomeric and filamentous proteins associated with apical, vascular and embryonic tissues in plants. *Plant Mol. Biol.* **55**, 579–594 (2004). 15604702 10.1007/s11103-004-1520-4

[7] Martinez, D. et al. Coiled-coil oligomerization controls localization of the plasma membrane REMORINs. *J. Struct. Biol.* **206**, 12–19 (2018). 29481850 10.1016/j.jsb.2018.02.003

[8] Perraki, A. et al. Plasma membrane localization of *Solanum tuberosum* remorin from group 1, homolog 3 is mediated by conformational changes in a novel C-terminal anchor and required for the restriction of potato virus X movement. *Plant Physiol.* **160**, 624–637 (2012). 22855937 10.1104/pp.112.200519 PMC3461544

[9] Legrand, A. et al. Structural determinants of REMORIN nanodomain formation in anionic membranes. *Biophys. J.* **122**, 2192–2202 (2022). 36582138 10.1016/j.bpj.2022.12.035 PMC10257120

[10] Legrand, A. et al. Nanodomain clustering of the plant protein remorin by solid-state NMR. *Front. Mol. Biosci.* **6**, 107 (2019). 31681795 10.3389/fmolb.2019.00107 PMC6803476

[11] Gouguet, P. et al. Connecting the dots: from nanodomains to physiological functions of REMORINs. *Plant Physiol.* **185**, 632–649 (2021). 33793872 10.1093/plphys/kiaa063 PMC8133660

[12] Gui, J., Liu, C., Shen, J. & Li, L. *Grain setting defect1*, encoding a remorin protein, affects the grain setting in rice through regulating plasmodesmatal conductance. *Plant Physiol.* **166**, 1463–1478 (2014). 25253885 10.1104/pp.114.246769 PMC4226345

[13] Bücherl, C. A. et al. Plant immune and growth receptors share common signalling components but localise to distinct plasma membrane nanodomains. *eLife* **6**, e25114 (2017). 28262094 10.7554/eLife.25114 PMC5383397

[14] Jarsch, I. K. et al. Plasma membranes are subcompartmentalized into a plethora of coexisting and diverse microdomains in *Arabidopsis* and *Nicotiana benthamiana*. *Plant Cell* **26**, 1698–1711 (2014). 24714763 10.1105/tpc.114.124446 PMC4036580

[15] Gronnier, J. et al. Structural basis for plant plasma membrane protein dynamics and organization into functional nanodomains. *elife* **6**, e26404 (2017). 28758890 10.7554/eLife.26404 PMC5536944

[16] Liang, P. et al. Symbiotic root infections in *Medicago truncatula* require remorin-mediated receptor stabilization in membrane nanodomains. *Proc. Natl Acad. Sci. USA* **115**, 5289–5294 (2018). 29712849 10.1073/pnas.1721868115 PMC5960310

[17] Demir, F. et al. Arabidopsis nanodomain-delimited ABA signaling pathway regulates the anion channel SLAH3. *Proc. Natl Acad. Sci. USA* **110**, 8296–8301 (2013). 23630285 10.1073/pnas.1211667110 PMC3657796

[18] Hemsley, P. A., Weimar, T., Lilley, K. S., Dupree, P. & Grierson, C. S. A proteomic approach identifies many novel palmitoylated proteins in *A**rabidopsis*. *New Phytol.* **197**, 805–814 (2013). 23252521 10.1111/nph.12077

[19] Konrad, S. S. A. et al. S‐acylation anchors remorin proteins to the plasma membrane but does not primarily determine their localization in membrane microdomains. *New Phytol.* **203**, 758–769 (2014). 24897938 10.1111/nph.12867

[20] Ma, T. et al. Palmitoylation is indispensable for remorin to restrict tobacco mosaic virus cell-to-cell movement in *Nicotiana benthamiana*. *Viruses* **14**, 1324 (2022). 35746795 10.3390/v14061324 PMC9227848

[21] Su, C. et al. Stabilization of membrane topologies by proteinaceous remorin scaffolds. *Nat. Commun.* **14**, 323 (2023). 36658193 10.1038/s41467-023-35976-5 PMC9852587

[22] Bailey, T. L., Johnson, J., Grant, C. E. & Noble, W. S. The MEME suite. *Nucleic Acids Res.* **43**, W39–W49 (2015). 25953851 10.1093/nar/gkv416 PMC4489269

[23] Jumper, J. et al. Highly accurate protein structure prediction with AlphaFold. *Nature* **596**, 583–589 (2021). 34265844 10.1038/s41586-021-03819-2 PMC8371605

[24] Mirdita, M. et al. ColabFold: making protein folding accessible to all. *Nat. Methods* **19**, 679–682 (2022). 35637307 10.1038/s41592-022-01488-1 PMC9184281

[25] Simm, D., Hatje, K., Waack, S. & Kollmar, M. Critical assessment of coiled-coil predictions based on protein structure data. *Sci. Rep.* **11**, 12439 (2021). 34127723 10.1038/s41598-021-91886-w PMC8203680

[26] Trigg, J., Gutwin, K., Keating, A. E. & Berger, B. Multicoil2: predicting coiled coils and their oligomerization states from sequence in the twilight zone. *PLoS ONE* **6**, e23519 (2011). 21901122 10.1371/journal.pone.0023519 PMC3162000

[27] Stevens, A. O. & He, Y. Benchmarking the accuracy of AlphaFold 2 in loop structure prediction. *Biomolecules* **12**, 985 (2022). 35883541 10.3390/biom12070985 PMC9312937

[28] Perraki, A. et al. REM1.3’s phospho-status defines its plasma membrane nanodomain organization and activity in restricting PVX cell-to-cell movement. *PLoS Pathog.* **14**, e1007378 (2018). 30419072 10.1371/journal.ppat.1007378 PMC6258466

[29] Brünger, A. T. et al. Crystallography & NMR system: a new software suite for macromolecular structure determination. *Acta Crystallogr. D. Biol. Crystallog.r* **54**, 905–921 (1998). 10.1107/s0907444998003254 9757107

[30] Shi, Y. et al. Polarizable atomic multipole-based AMOEBA force field for proteins. *J. Chem. Theory Comput.* **9**, 4046–4063 (2013). 24163642 10.1021/ct4003702 PMC3806652

[31] Wang, Y. & Jardetzky, O. Probability-based protein secondary structure identification using combined NMR chemical-shift data. *Protein Sci.* **11**, 852–861 (2002). 11910028 10.1110/ps.3180102 PMC2373532

[32] Kirsh, J. M., Weaver, J. B., Boxer, S. G. & Kozuch, J. Critical evaluation of polarizable and nonpolarizable force fields for proteins using experimentally derived nitrile electric fields. *J. Am. Chem. Soc.* **146**, 6983–6991 (2024). 38415598 10.1021/jacs.3c14775 PMC10941190

[33] Pauling, L. & Corey, R. B. Compound helical configurations of polypeptide chains: structure of proteins of the α-keratin type. *Nature* **171**, 59–61 (1953). 13025480 10.1038/171059a0

[34] Lupas, A. Coiled coils: new structures and new functions. *Trends Biochem. Sci.* **21**, 375–382 (1996). 8918191

[35] Crick, F. H. C. Is α-keratin a coiled coil? *Nature* **170**, 882–883 (1952). 13013241 10.1038/170882b0

[36] Madeo, G., Savojardo, C., Manfredi, M., Martelli, P. L. & Casadio, R. CoCoNat: a novel method based on deep learning for coiled-coil prediction. *Bioinformatics* **39**, btad495 (2023). 37540220 10.1093/bioinformatics/btad495 PMC10425188

[37] Bottaro, S. & Lindorff-Larsen, K. Biophysical experiments and biomolecular simulations: a perfect match? *Science* **361**, 355–360 (2018). 30049874 10.1126/science.aat4010

[38] Case, D. A. et al. The Amber biomolecular simulation programs. *J. Comput Chem.* **26**, 1668–1688 (2005). 16200636 10.1002/jcc.20290 PMC1989667

[39] Jackson, V. et al. The guidance and adhesion protein FLRT2 dimerizes in cis via dual small-X3-small transmembrane motifs. *Structure* **30**, 1354–1365.e5 (2022). 35700726 10.1016/j.str.2022.05.014

[40] Badaczewska-Dawid, A. E., Nithin, C., Wroblewski, K., Kurcinski, M. & Kmiecik, S. MAPIYA contact map server for identification and visualization of molecular interactions in proteins and biological complexes. *Nucleic Acids Res.* **50**, W474–W482 (2022). 35524560 10.1093/nar/gkac307 PMC9252833

[41] Redfern, D. A. & Gericke, A. Domain formation in phosphatidylinositol monophosphate/phosphatidylcholine mixed vesicles. *Biophys. J.* **86**, 2980–2992 (2004). 15111413 10.1016/S0006-3495(04)74348-9 PMC1304165

[42] Motegi, T., Takiguchi, K., Tanaka-Takiguchi, Y., Itoh, T. & Tero, R. Physical properties and reactivity of microdomains in phosphatidylinositol-containing supported lipid bilayer. *Membranes* **11**, 339 (2021). 34063660 10.3390/membranes11050339 PMC8147626

[43] Ruff, K. M. & Pappu, R. V. AlphaFold and implications for intrinsically disordered proteins. *J. Mol. Biol.* **433**, 167208 (2021). 34418423 10.1016/j.jmb.2021.167208

[44] Tesei, G. et al. Conformational ensembles of the human intrinsically disordered proteome. *Nature* **626**, 897–904 (2024). 38297118 10.1038/s41586-023-07004-5

[45] Kumari, P. et al. Structural insights into α-synuclein monomer–fibril interactions. *Proc. Natl Acad. Sci. USA* **118**, e2012171118 (2021). 33649211 10.1073/pnas.2012171118 PMC7958257

[46] Faidon Brotzakis, Z. et al. Determination of the structure and dynamics of the fuzzy coat of an amyloid fibril of IAPP using cryo-electron microscopy. *Biochemistry* **62**, 2407–2416 (2023). 37477459 10.1021/acs.biochem.3c00010 PMC10433526

[47] Ulamec, S. M., Brockwell, D. J. & Radford, S. E. Looking beyond the core: the role of flanking regions in the aggregation of amyloidogenic peptides and proteins. *Front. Neurosci.* **14**, 611285 (2020). 33335475 10.3389/fnins.2020.611285 PMC7736610

[48] Wegmann, S., Medalsy, I. D., Mandelkow, E. & Müller, D. J. The fuzzy coat of pathological human Tau fibrils is a two-layered polyelectrolyte brush. *Proc. Natl Acad. Sci. USA* **110**, E313–E321 (2013). 10.1073/pnas.1212100110 PMC3557036 23269837

[49] Holm, L., Laiho, A., Törönen, P. & Salgado, M. DALI shines a light on remote homologs: one hundred discoveries. *Protein Sci.* **32**, e4519 (2023). 36419248 10.1002/pro.4519 PMC9793968

[50] Holm, L. DALI and the persistence of protein shape. *Protein Sci.* **29**, 128–140 (2020). 31606894 10.1002/pro.3749 PMC6933842

[51] Wayment-Steele, H. K. et al. Predicting multiple conformations via sequence clustering and AlphaFold2. *Nature* **625**, 832–839 (2024). 37956700 10.1038/s41586-023-06832-9 PMC10808063

[52] Rahmani, H. et al. The myosin II coiled-coil domain atomic structure in its native environment. *Proc. Natl Acad. Sci. USA* **118**, e2024151118 (2021). 33782130 10.1073/pnas.2024151118 PMC8040620

[53] Lou, X. & Shin, Y.-K. SNARE zippering. *Biosci. Rep.* **36**, e00327 (2016). 27154457 10.1042/BSR20160004 PMC4859083

[54] Marín, M., Thallmair, V. & Ott, T. The intrinsically disordered N-terminal region of AtREM1.3 remorin protein mediates protein-protein interactions. *J. Biol. Chem.* **287**, 39982–39991 (2012). 23027878 10.1074/jbc.M112.414292 PMC3501056

[55] Alderson, T. R., Pritišanac, I., Kolarić, Đ., Moses, A. M. & Forman-Kay, J. D. Systematic identification of conditionally folded intrinsically disordered regions by AlphaFold2. *Proc. Natl Acad. Sci. USA* **120**, e2304302120 (2023). 37878721 10.1073/pnas.2304302120 PMC10622901

[56] Yoshizawa, T., Nozawa, R.-S., Jia, T. Z., Saio, T. & Mori, E. Biological phase separation: cell biology meets biophysics. *Biophys. Rev.* **12**, 519–539 (2020). 32189162 10.1007/s12551-020-00680-x PMC7242575

[57] Sievers, F. et al. Fast, scalable generation of high‐quality protein multiple sequence alignments using clustal omega. *Mol. Syst. Biol.* **7**, 539 (2011). 21988835 10.1038/msb.2011.75 PMC3261699

[58] Altschul, S. F., Gish, W., Miller, W., Myers, E. W. & Lipman, D. J. Basic local alignment search tool. *J. Mol. Biol.* **215**, 403–410 (1990). 2231712 10.1016/S0022-2836(05)80360-2

[59] Mirdita, M., Steinegger, M. & Söding, J. MMseqs2 desktop and local web server app for fast, interactive sequence searches. *Bioinformatics* **35**, 2856–2858 (2019). 30615063 10.1093/bioinformatics/bty1057 PMC6691333

[60] Schanda, P. & Brutscher, B. Very fast two-dimensional NMR spectroscopy for real-time investigation of dynamic events in proteins on the time scale of seconds. *J. Am. Chem. Soc.* **127**, 8014–8015 (2005). 15926816 10.1021/ja051306e

[61] Hwang, T. L. & Shaka, A. J. Water suppression that works. excitation sculpting using arbitrary wave-forms and pulsed-field gradients. *J. Magn. Reson. Ser. A* **112**, 275–279 (1995).

[62] Shaka, A. J., Lee, C. J. & Pines, A. Iterative schemes for bilinear operators; application to spin decoupling. *J. Magn. Reson.* **77**, 274–293 (1988).

[63] Bockmann, A. et al. Characterization of different water pools in solid-state NMR protein samples. *J. Biomol. NMR* **45**, 319–327 (2009). 19779834 10.1007/s10858-009-9374-3

[64] Vranken, W. F. et al. The CCPN data model for NMR spectroscopy: development of a software pipeline. *Proteins* **59**, 687–696 (2005). 15815974 10.1002/prot.20449

[65] Allain, F., Mareuil, F., Ménager, H., Nilges, M. & Bardiaux, B. ARIAweb: a server for automated NMR structure calculation. *Nucleic Acids Res.* **48**, W41–W47 (2020). 32383755 10.1093/nar/gkaa362 PMC7319541

[66] Hornak, V. et al. Comparison of multiple Amber force fields and development of improved protein backbone parameters. *Proteins* **65**, 712–725 (2006). 16981200 10.1002/prot.21123 PMC4805110

[67] Maier, J. A. et al. ff14SB: improving the accuracy of protein side chain and backbone parameters from ff99SB. *J. Chem. Theory Comput.* **11**, 3696–3713 (2015). 26574453 10.1021/acs.jctc.5b00255 PMC4821407

[68] Abraham, M. J. et al. GROMACS: high performance molecular simulations through multi-level parallelism from laptops to supercomputers. *SoftwareX* **1–2**, 19–25 (2015).

[69] Lemkul, J. From proteins to perturbed Hamiltonians: a suite of tutorials for the GROMACS-2018 molecular simulation package [Article v1.0]. *LiveCoMS* **1**, 5068 (2019).

[70] Olsson, M. H. M., Søndergaard, C. R., Rostkowski, M. & Jensen, J. H. PROPKA3: consistent treatment of internal and surface residues in empirical p *K*_a_ predictions. *J. Chem. Theory Comput.* **7**, 525–537 (2011). 26596171 10.1021/ct100578z

[71] Lagardère, L. et al. Tinker-HP: a massively parallel molecular dynamics package for multiscale simulations of large complex systems with advanced point dipole polarizable force fields. *Chem. Sci.* **9**, 956–972 (2018). 29732110 10.1039/c7sc04531j PMC5909332

[72] Tian, C. et al. ff19SB: amino-acid-specific protein backbone parameters trained against quantum mechanics energy surfaces in solution. *J. Chem. Theory Comput.* **16**, 528–552 (2020). 31714766 10.1021/acs.jctc.9b00591 PMC13071887

[73] Lagardère, L., Aviat, F. & Piquemal, J.-P. Pushing the limits of multiple-time-step strategies for polarizable point dipole molecular dynamics. *J. Phys. Chem. Lett.* **10**, 2593–2599 (2019). 31050904 10.1021/acs.jpclett.9b00901

[74] Jaffrelot Inizan, T. et al. High-resolution mining of the SARS-CoV-2 main protease conformational space: supercomputer-driven unsupervised adaptive sampling. *Chem. Sci.* **12**, 4889–4907 (2021). 34168762 10.1039/d1sc00145k PMC8179654

[75] Pedregosa, F. et al. Scikit-learn: Machine Learning in Python. Preprint at 10.48550/ARXIV.1201.0490 v4 (2012).

[76] McGibbon, R. T. et al. MDTraj: a modern open library for the analysis of molecular dynamics trajectories. *Biophys. J.* **109**, 1528–1532 (2015). 26488642 10.1016/j.bpj.2015.08.015 PMC4623899

[77] Virtanen, P. et al. SciPy 1.0: fundamental algorithms for scientific computing in Python. *Nat. Methods* **17**, 261–272 (2020). 32015543 10.1038/s41592-019-0686-2 PMC7056644

[78] Zacharias, D. A., Violin, J. D., Newton, A. C. & Tsien, R. Y. Partitioning of lipid-modified monomeric GFPs into membrane microdomains of live cells. *Science* **296**, 913–916 (2002). 11988576 10.1126/science.1068539

