# Supplementary material for: Dynamic pre-structuration of lipid nanodomain-segregating remorin proteins
Source: Commun Biol. 2024 Dec 5;7:1620. doi: 10.1038/s42003-024-07330-y (PMC11621693; doi:10.1038/s42003-024-07330-y)
Supplement: Supplementary file 1 — Supplementary Information [file 42003_2024_7330_MOESM1_ESM.pdf]

Supplementary Information

Dynamic pre-structuration of lipid nanodomain-segregating remorin proteins

AUTHORS: Zeren Xu[1], Adrien Schahl[2,3], Marie-Dominique Jolivet[4], Anthony Legrand[1], Axelle Grélard[1], Mélanie Berbon[1], Estelle Morvan[5], Louis Lagardere[3], Jean-Philip Piquemal[3], Antoine Loquet[1], Véronique Germain[4], Matthieu Chavent[2,6#], Sébastien Mongrand[4#], Birgit Habenstein[1#]

AFFILIATIONS

1 Univ. Bordeaux, CNRS, Bordeaux INP, CBMN, UMR 5248, IECB, F-33600 Pessac, France

2 Institut de Pharmacologie et de Biologie Structurale, Université de Toulouse, CNRS, Université Paul Sabatier, 31400 Toulouse, France

3 Sorbonne Université, LCT, UMR7616 CNRS,75005Paris, France; Qubit Pharmaceuticals, Advanced Research Department, 75014 Paris, France

4 Laboratoire de Biogenèse Membranaire (LBM) UMR-5200, CNRS-Univ. Bordeaux, F-33140 Villenave d'Ornon, France

5 Univ. Bordeaux, CNRS, Inserm, IECB, UAR3033, US01, Pessac, France

6 Laboratoire de Microbiologie et Génétique Moléculaires (LMGM), Centre de Biologie Intégrative (CBI), Université de Toulouse, CNRS, UPS, France

# corresponding author: M.C. Matthieu.Chavent@ipbs.fr, S.M. sebastien.mongrand@u-bordeaux.fr, B.H. b.habenstein@cbmn.u-bordeaux.fr

§ A.S. now works at Department of Applied Physics, Science for Life Laboratory, Royal Institute of Technology, Solna SE-171 21, Sweden; M.D.L. now works at Center for Plant Molecular Biology (ZMBP), University of Tübingen, Germany; A.Le. now works Loschmidt Laboratories, Masaryk University, Brno, Czechia.

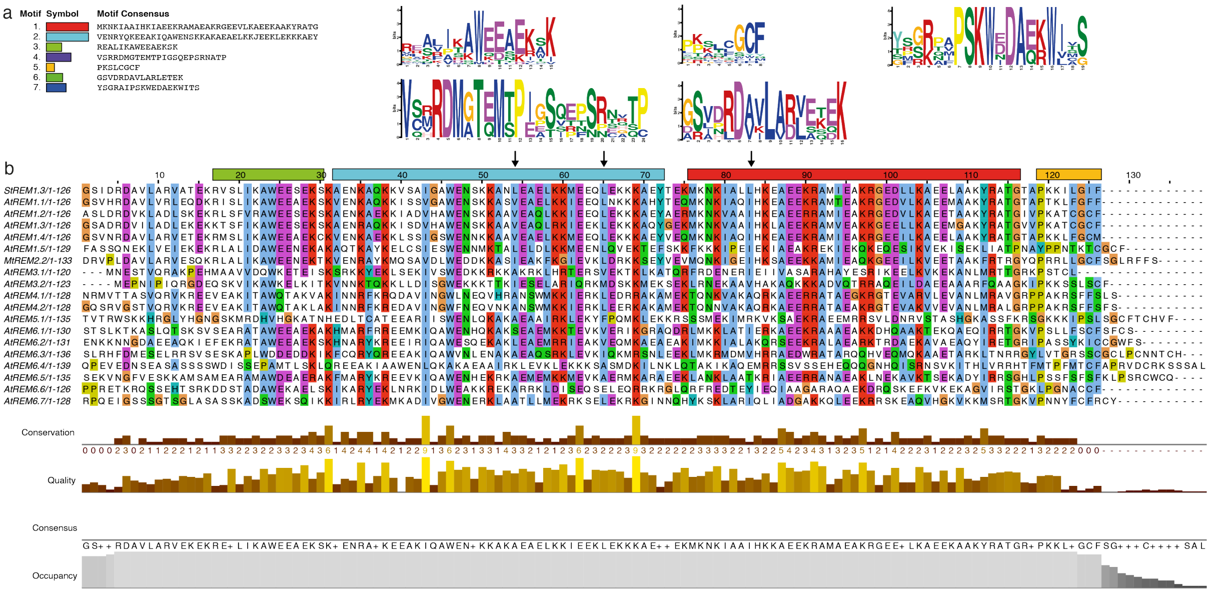

Supplementary Figure 1: Amino acid sequence conservation within the Remorin family[1]. a Sequence motif conservation in bits based on the MEME suite[2]. b Sequence alignment and visualizing by Blast (NCBI) and Clustal Omega, respectively.[3,4]

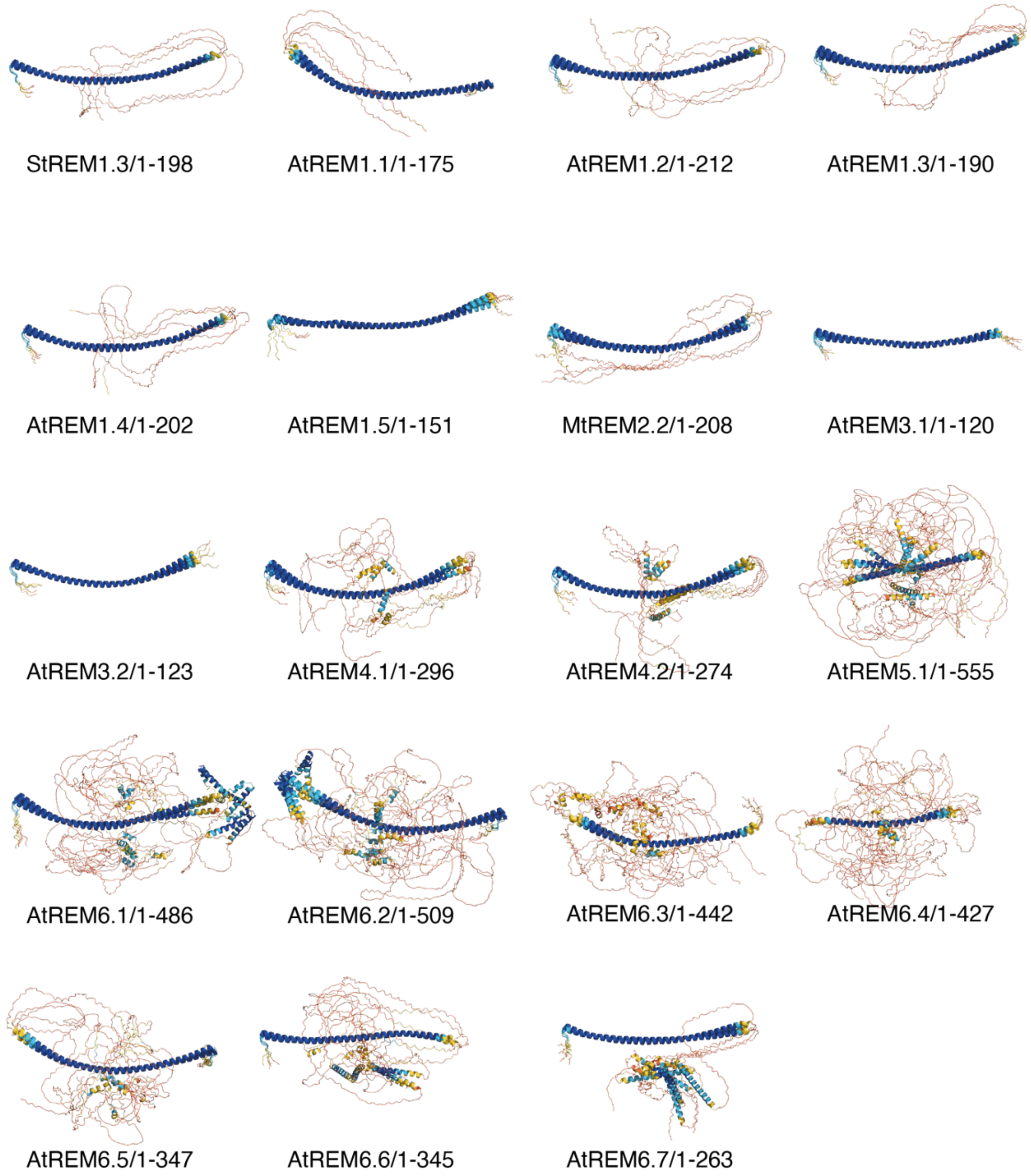

Supplementary Figure 2: Ensemble of 5 monomeric structures of remorin family members predicted by Alphafold 2[5] and colored dependent on the pLDDT score (blue: pLDDT > 90; cyan 90 > pLDDT > 70; yellow 70 > pLDDT > 50; red pLDDT < 50, see also Figure 1 in the main text).

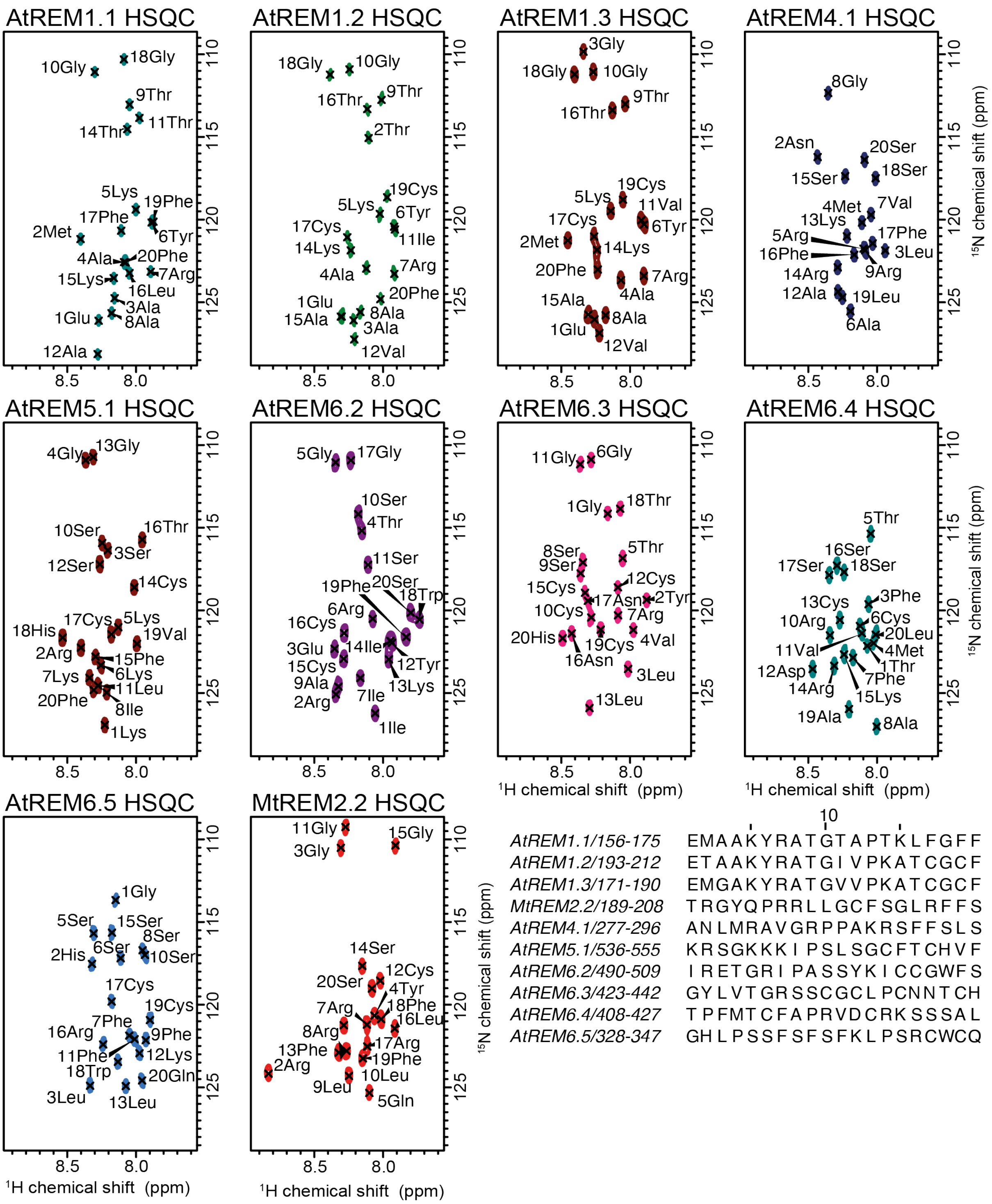

Supplementary Figure 3: 2D ^{1}H-^{15}N solution NMR spectra members recorded at 800MHz using the SOFAST-HMQC pulse sequence of 10 selected REM-CA sequences of remorin family members, as detailed in the lower right panel. The residue-specific assignments of the ^{1}H and ^{15}N chemical shifts are indicated on the spectra. Residues are numbered as indicated in the lower right panel.

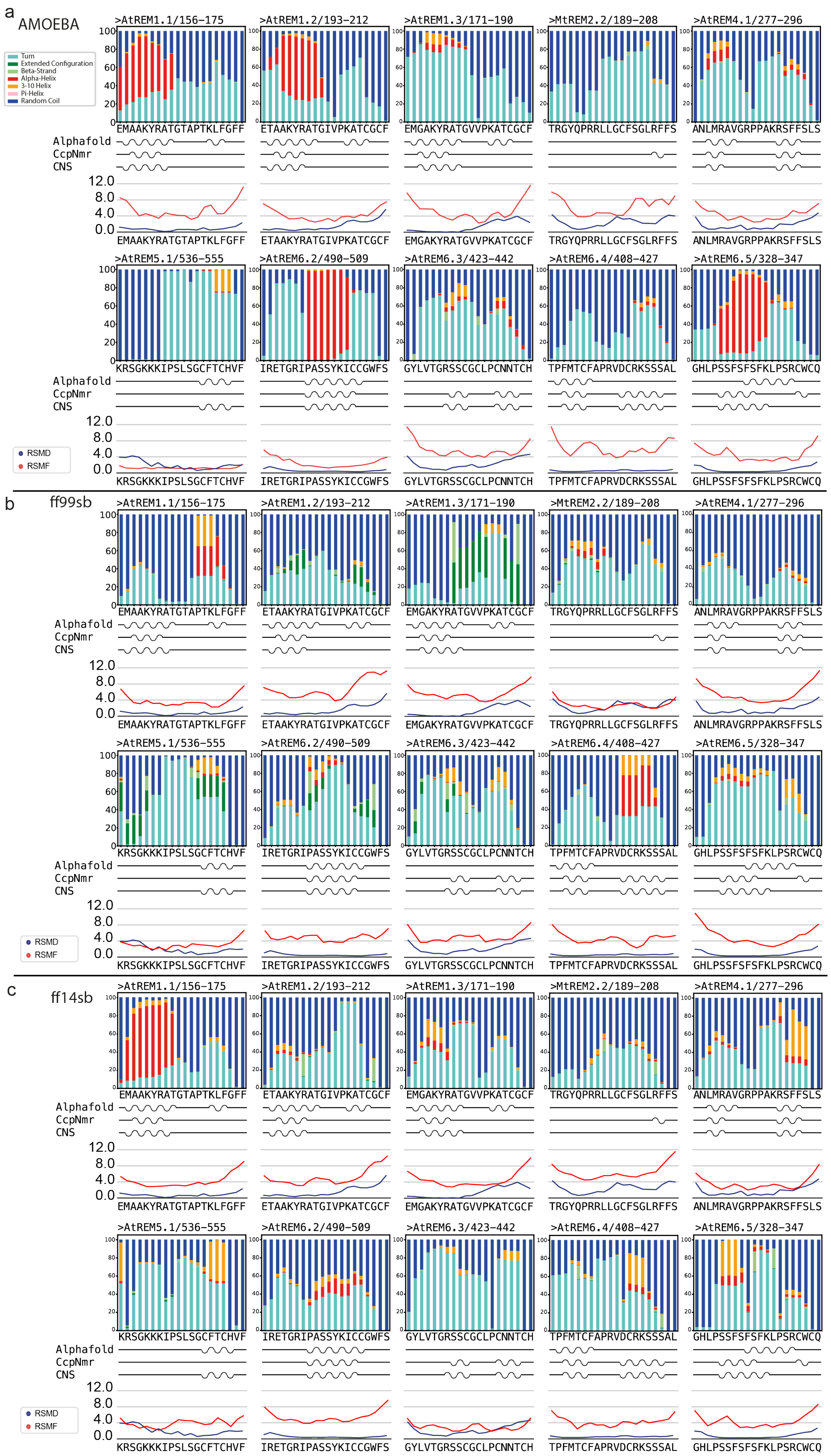
a
AMOEBA
b
ff99sb
c
ff14sb
Turn
Extended Configuration
Beta-Strand
Alpha-Helix
3-10 Helix
Pi-Helix
Random Coil
RSMD
RSMF
Alphafold
CcpNmr
CNS
>AtREM1.1/156–175
>AtREM1.2/193–212
>AtREM1.3/171–190
>MtREM2.2/189–208
>AtREM4.1/277–296
>AtREM5.1/536–555
>AtREM6.2/490–509
>AtREM6.3/423–442
>AtREM6.4/408–427
>AtREM6.5/328–347
EMAAKYRATGTAPTKLFGFF
ETAAKYRATGIVPKATCGCF
EMGAKYRATGVVPKATCGCF
TRGYQPRRLLGCFSGLRFFS
ANLMRAVGRPPAKRSFFSLS
KRSGKKKIPSLSGCFTCHVF
IRETGRIPASSYKICCGWFS
GYLVTGRSSCGCLPCNNTCH
TPFMTCFAPRVDCRKSSSAL
GHLPSSFSFSFKLPSRCWCQ
12.0
8.0
4.0
0.0

Supplementary Figure 4: Structure propensity of REM-CA structures simulated with atomistic molecular dynamics simulations using the AMOEBA, the AMBER ff99sb and the AMBER ff14sb forcefield over 1 µs respectively. The color code for structural motifs is indicated on the upper left panel. Indicated below is a cartoon of the secondary structure as determined by AF2 performed on the respective REM-CA, CcpNMR analysis[6], CNS structure calculation [7], the R.M.S.F of MD simulations and the R.M.S.D of the NMR structures.

```
                          10        20        30        40
StREM1.3/150-198   NKIALLHKEAEEKRAMIEAKRGEDLLKAEELAAKYRATGTAPKKILGIF
StREM1.3/160-198   ----------EEKRAMIEAKRGEDLLKAEELAAKYRATGTAPKKILGIF
StREM1.3/171-198   ---------------------GEDLLKAEELAAKYRATGTAPKKILGIF
```

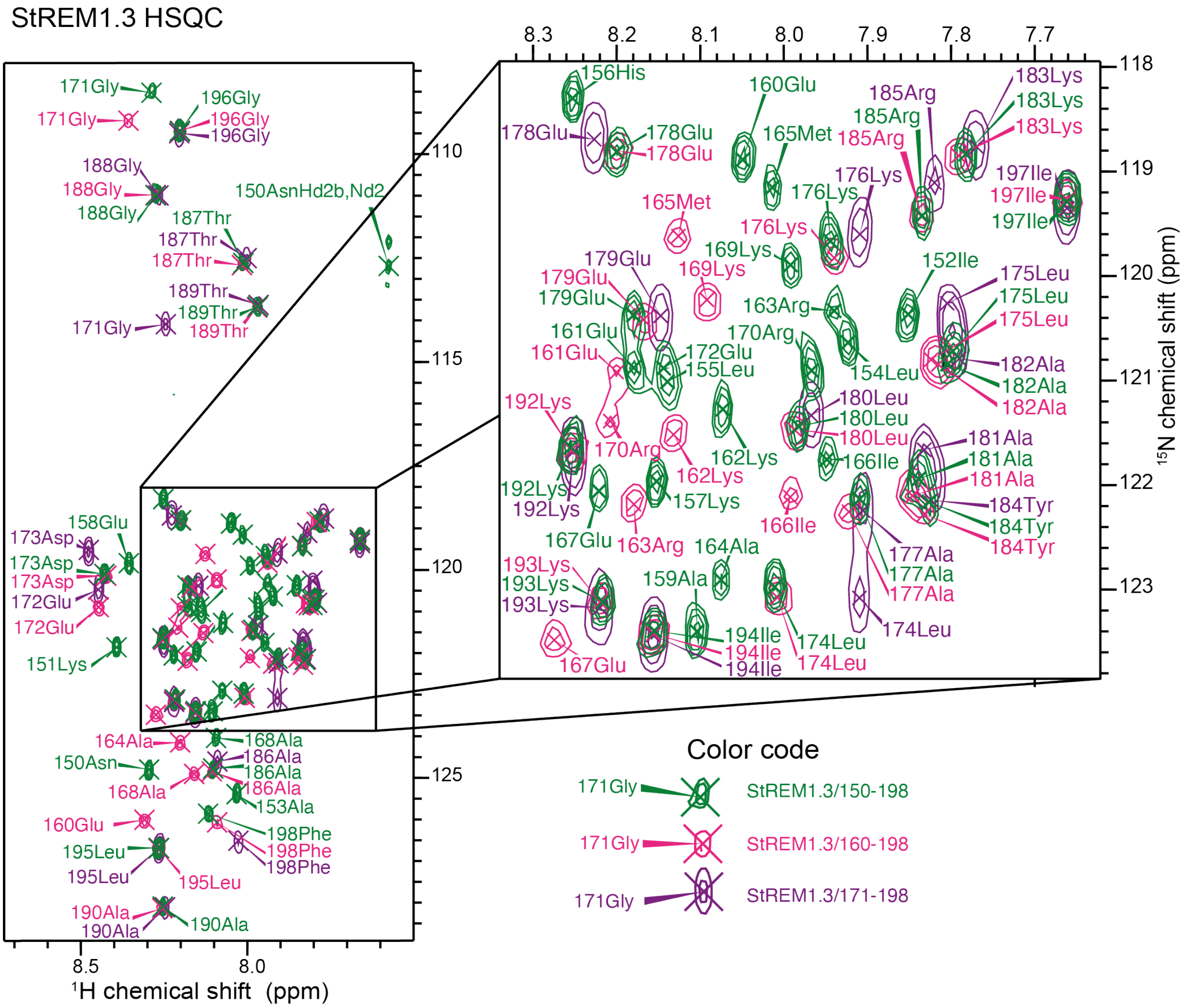

Supplementary Figure 5: 2D ^{1}H-^{15}N SOFAST-HMQC solution NMR spectra of 3 extended REM-CA sequences, namely StREM$_{150\text{-}198}$ (green), StREM$_{160\text{-}198}$ (pink) and StREM$_{171\text{-}198}$ (purple). The primary sequences of the three StREM sequences are denoted above. Residue-specific chemical shift assignment of extended StREM REM-CAs is indicated on the spectra with the color code specified in the lower right panel.

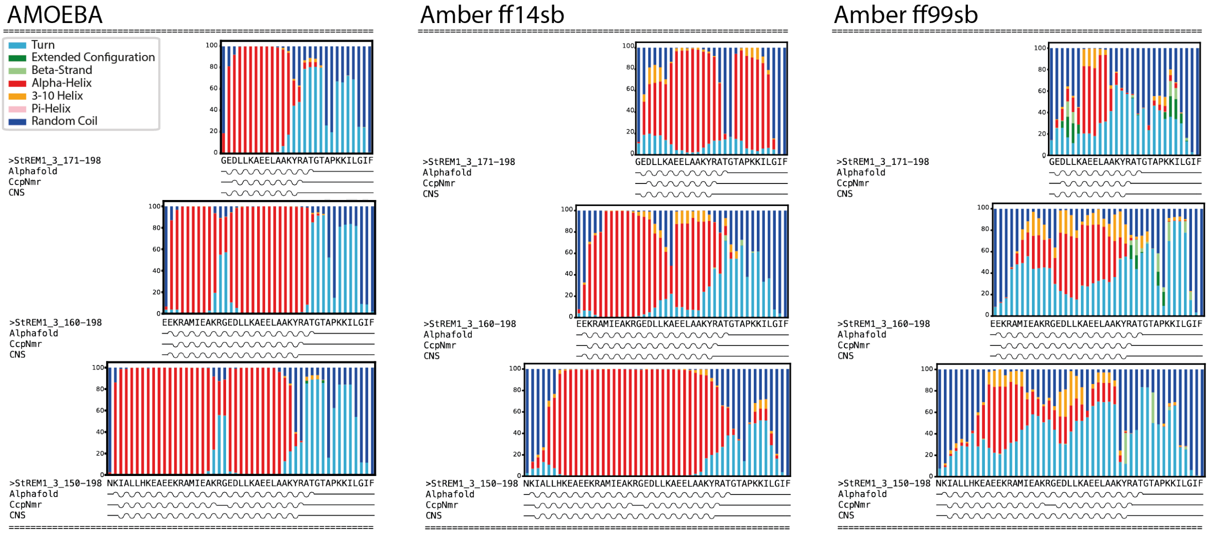

Supplementary Figure 6: Structure propensity of extended REM-CA structures of StREM$_{150-198}$, StREM$_{160-198}$ and StREM$_{171-198}$ simulated with atomistic molecular dynamics simulations using the AMOEBA, the AMBER ff99sb and the AMBER ff14sb forcefield over 1 μs respectively. The color code for structural motifs is indicated on the upper left panel. Indicated below is a cartoon of the secondary structure as determined by AF2 performed on the respective extended REM-CA, CcpNMR analysis[6] and CNS structure calculation [7].

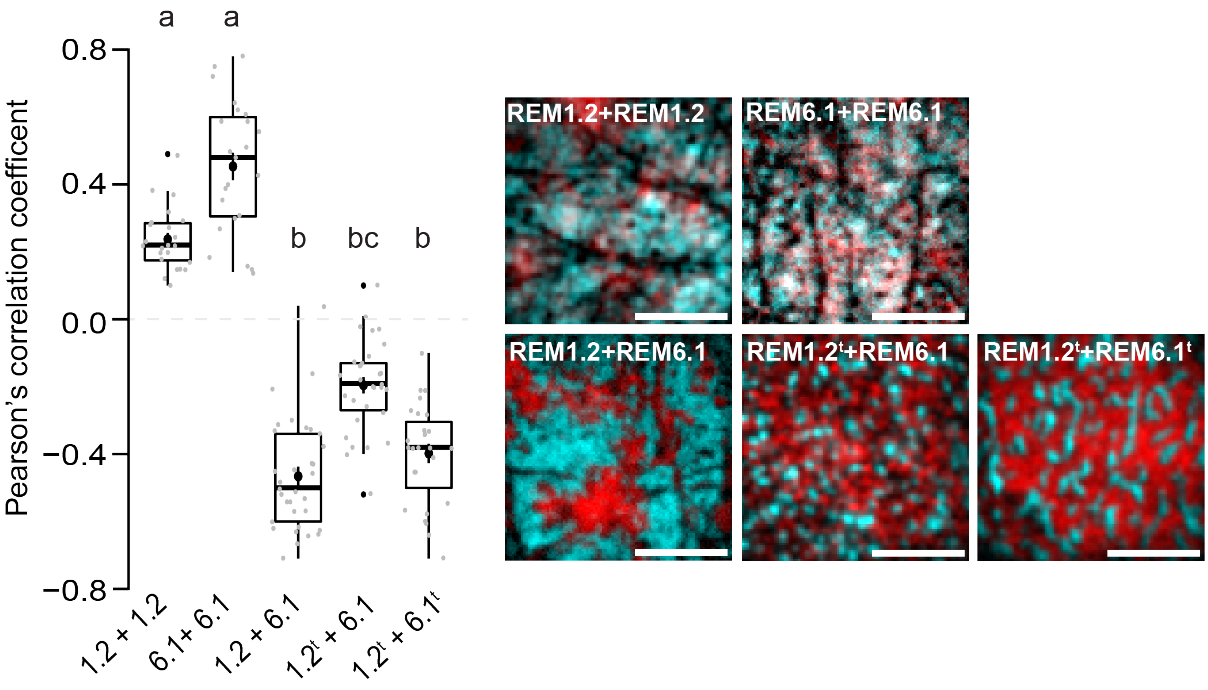

Supplementary Figure 7: The pearson's correlation coefficient (left panel) of full-length or truncated REM1.2 and/or REM6.1 was calculated from at least 23 cells over the course of 3 independent experiments. Significant differences were determined using a Kruskal-Wallis test followed by a Dunn's multiple comparison test. Different letters indicate significant differences ($p>0.01$). Scale bar = 4μm. Representative dual-color TIRF images (right panel) of the surface of epidermal cells of *Nicotiana benthamiana* transiently co-expressing full-length or truncated

AtREM1.2 and/or AtREM6.1, labeled with mRFP1.2 (red) or mVenus (cyan), with AtREM1.2 in red and AtREM6.1 in cyan when co-expressed.

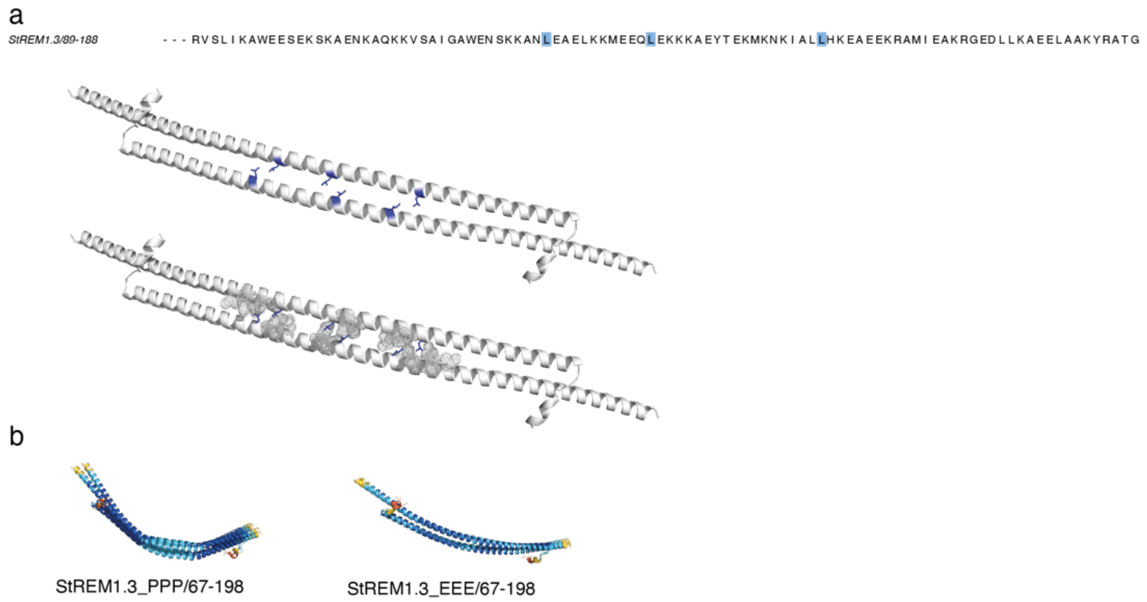

Supplementary Figure 8: a C-terminal region of StREM1.3 including the coiled-coil domain. Indicated in blue on the primary sequence and on the AF2 predicted dimeric structure are the mutated L126, L137 and L155 (upper panel) with the surfaces of the interfacing residues providing the knob-into-hole arrangement (lower panel). b Ensemble (5 structures) of AF2 predictions of $StREM_{67\text{-}198\ PPP}$ and $StREM_{67\text{-}198\ EEE}$ dimers, containing the replaced residues (L126, L137 and L155 to Pro or Glu, respectively) in the AF2 structure prediction. The color code represents the pLDDT score (blue: pLDDT > 90; cyan 90 > pLDDT > 70; yellow 70 > pLDDT > 50; red pLDDT < 50, see also Figure 1 in the main text).

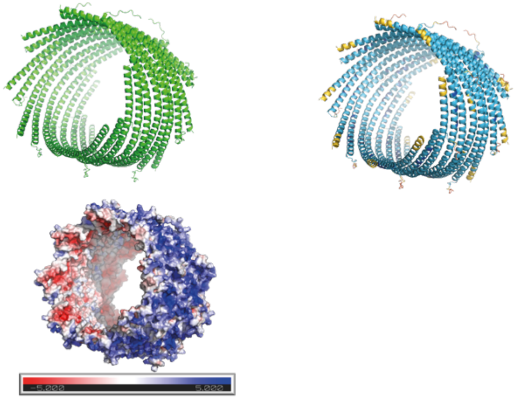

StREM1.3/67-198 16 multimer

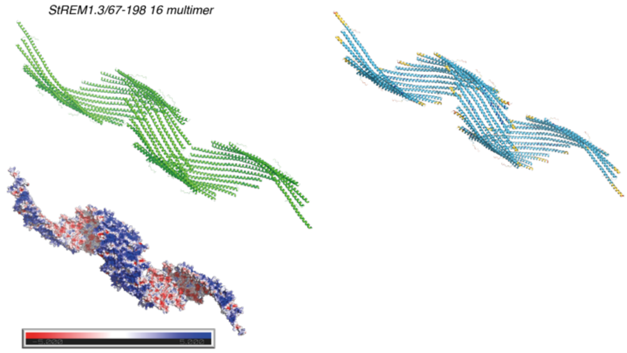

StREM1.3/67-198 32 multimer

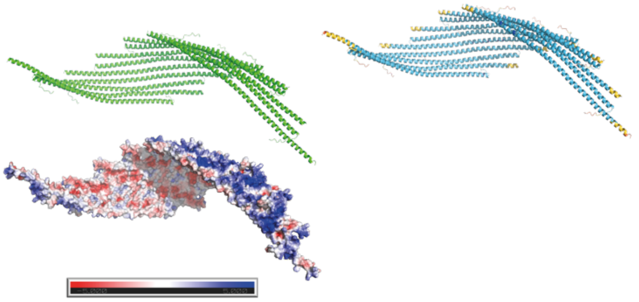

StREM1.3/67-198 12-Mers

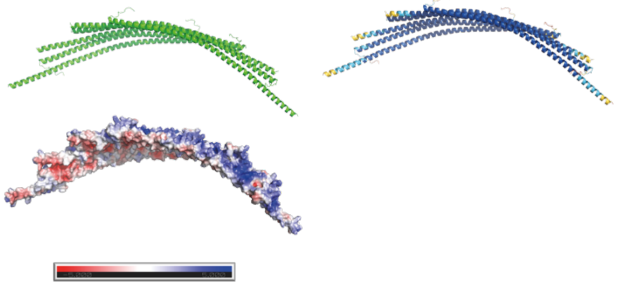

StREM1.3/67-198 6-Mers

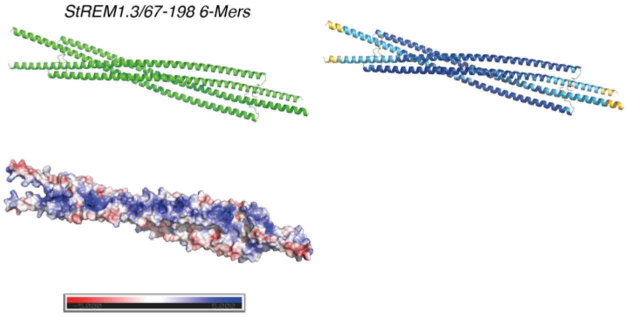

StREM1.3/67-198 4-Mers

Supplementary Figure 9: AF2 Multimer prediction containing a different number of the StREM$_{67\text{-}198\ \mathrm{PPP}}$ C-terminal region for each prediction. Structures and compositions are denoted below each prediction, and for each prediction are depicted the structure in cartoon (upper left panel), colored dependent on the pLDDT score, and in surface representation colored by charge distribution (red = negative charges, blue = positive charges). The color code represents the pLDDT score (blue: pLDDT > 90; cyan 90 > pLDDT > 70; yellow 70 > pLDDT > 50; red pLDDT < 50, see also Figure 1 in the main text).

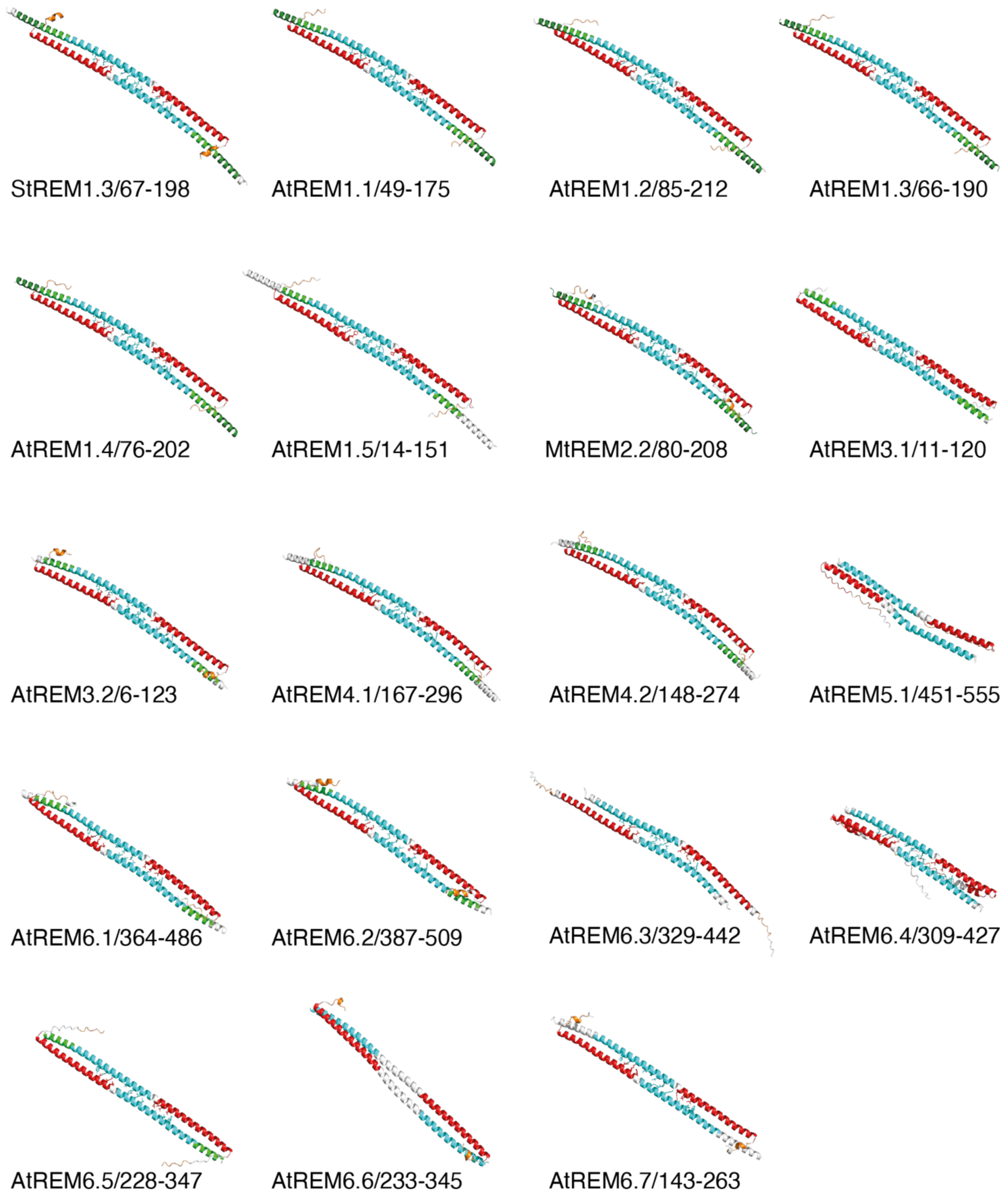

Supplementary Figure 10: Motif 1 and 2 highlighted on AF2-predicted dimeric structures for the C-terminal region of REM proteins from REM groups 1-6.

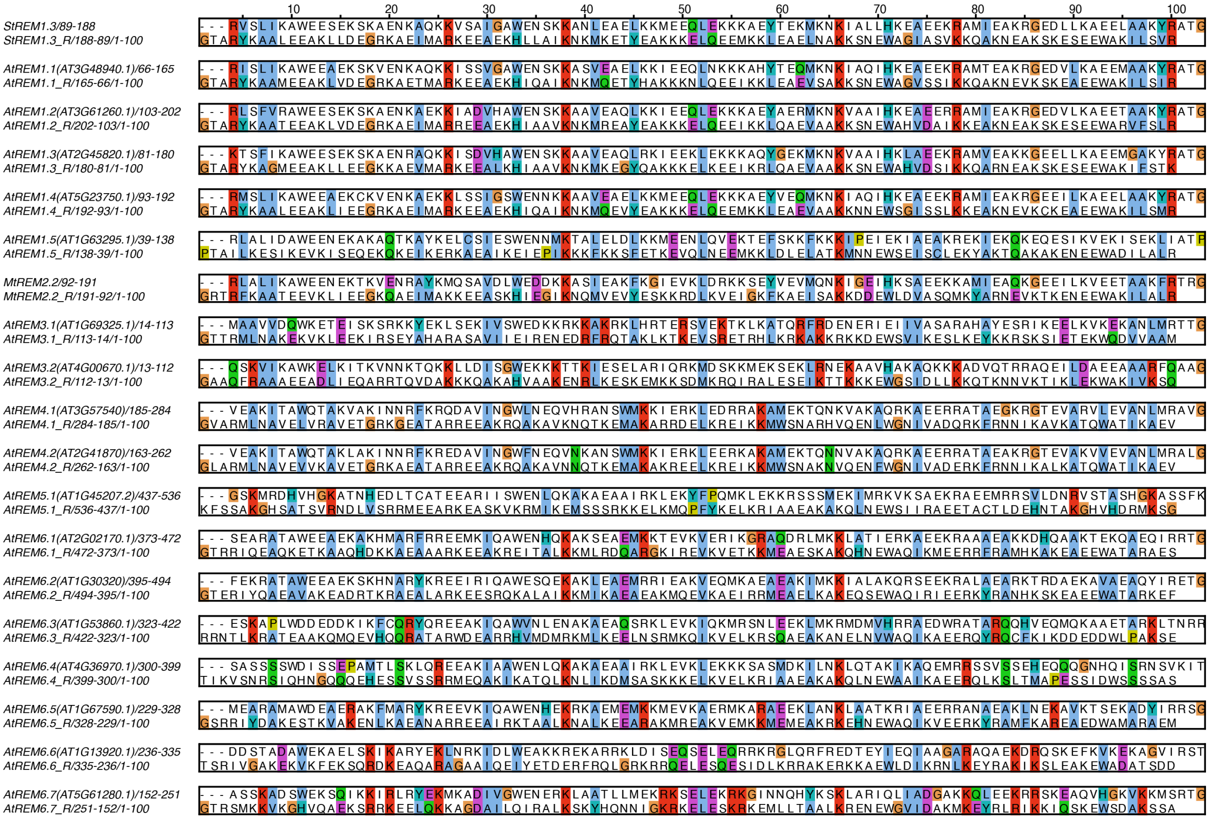

Supplementary Figure 11: Inverse alignment of the coil-coil domains centered on the symmetry axis (L137 in StREM1.3). Inverse alignment of the coiled-coil region using Clustal Omega [3] shows potential sources of different multimerization states and parallel *versus* anti-parallel coiled-coil arrangement.

StREM1.3/67-198_Dimer_All-Atom

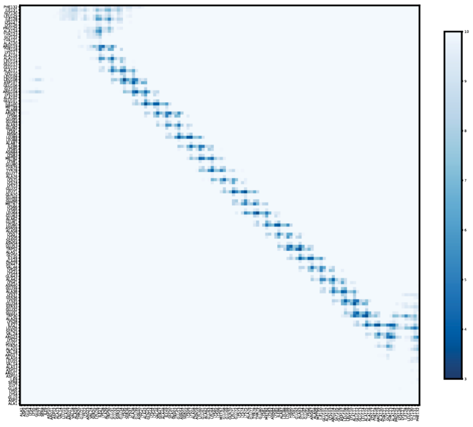

AtREM1.2/85-212_Dimer_All-Atom

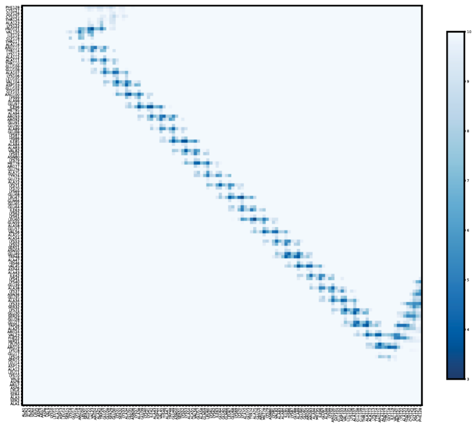

AtREM4.2/148-274_Dimer_All-Atom

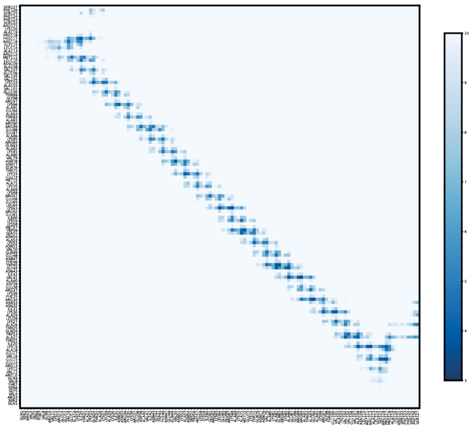

AtREM5.1/451-555_Dimer_All-Atom

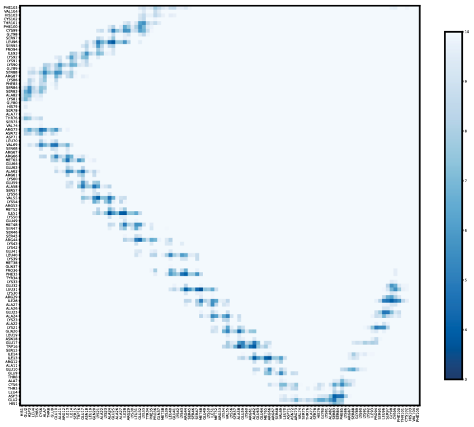

AtREM6.1/364-486_Dimer_All-Atom

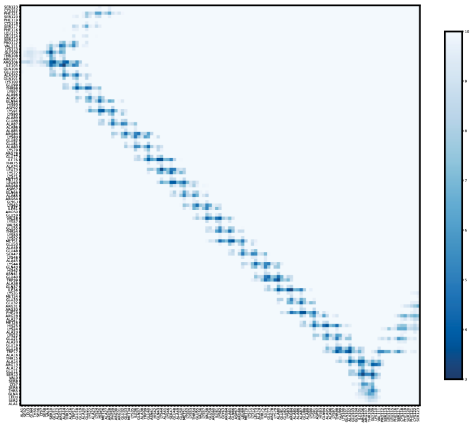

AtREM6.4/309-427_Dimer_All-Atom

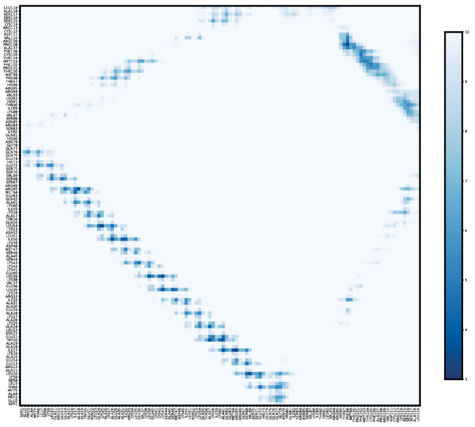

AtREM6.6/233-345_Dimer_All-Atom

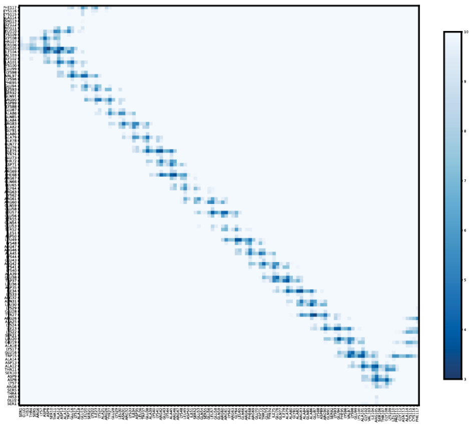

MtREM2.2/80-208_Dimer_All-Atom

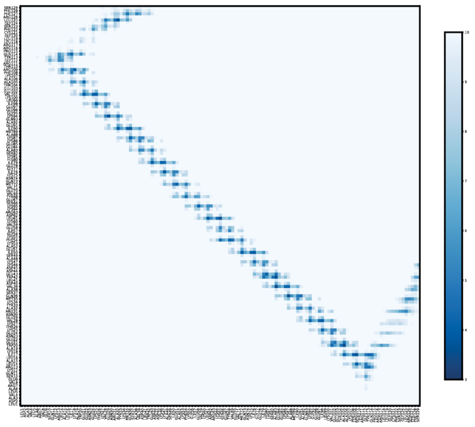

Supplementary Figure 12: Intermolecular contact map of Cα of monomer 1 and all atoms of monomer 2 (range 3-10 Å) of the structures adopted throughout the 1 μs atomistic MD simulation based on the Amber forcefield[8]. The blue scale is detailed in the right colorbars.

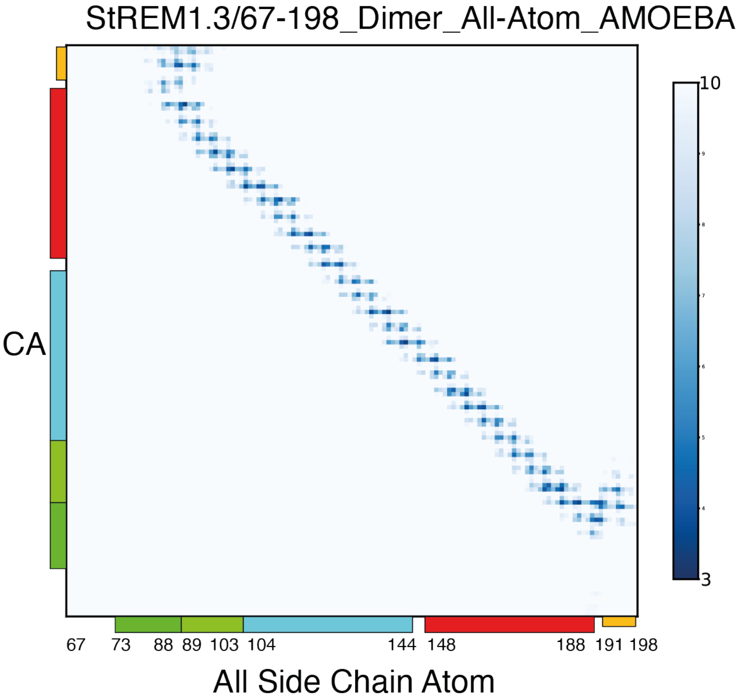

Supplementary Figure 13: Intermolecular contact map of Cα of monomer 1 and all atoms of monomer 2 (range 3-10 Å) of the structures adopted throughout the 560 ns atomistic MD simulation based on the AMOEBA forcefield. The blue scale is detailed in the right colorbar and as in Supplementary Figure 13.

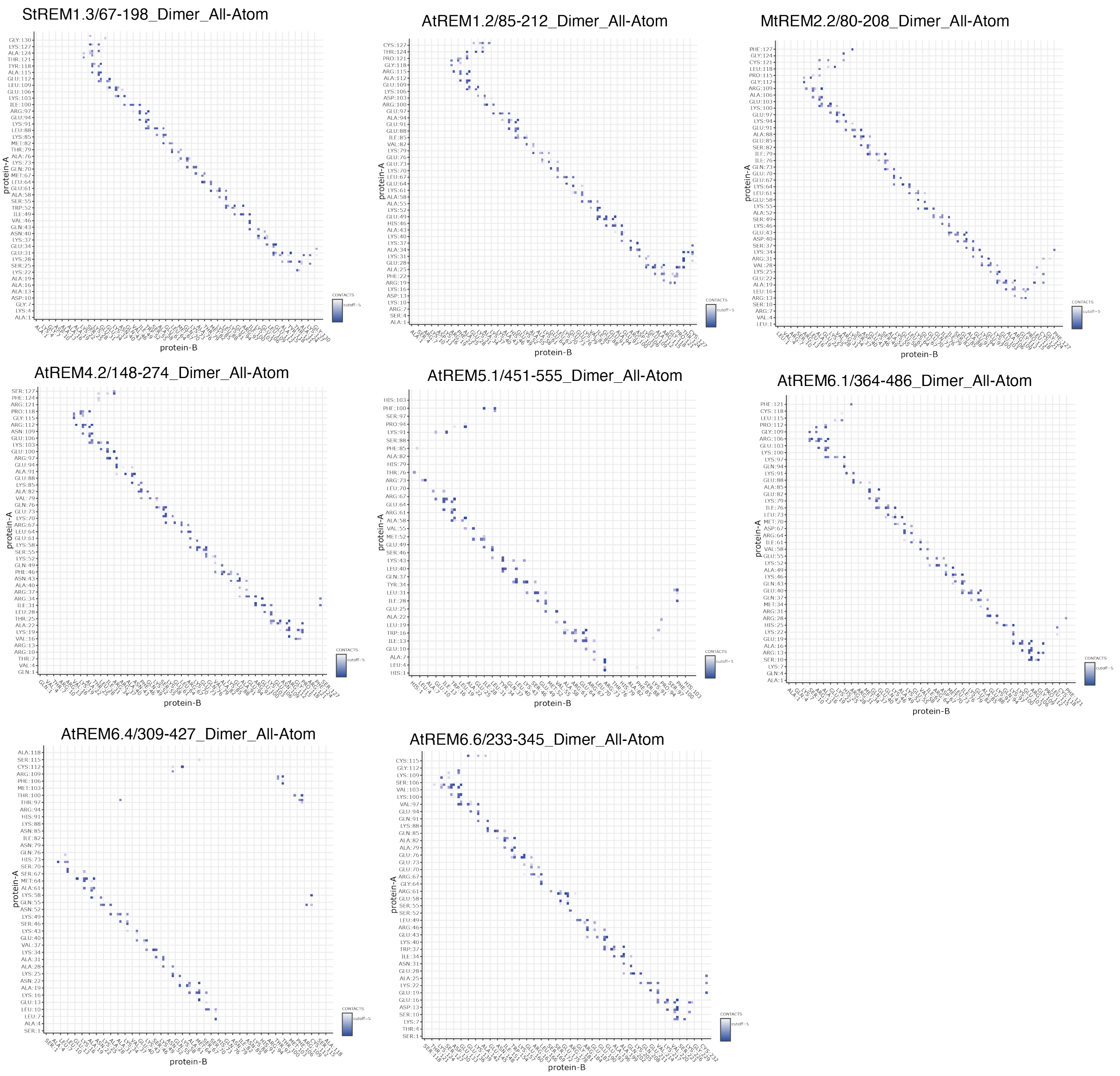

Supplementary Figure 14: Interatomic all-atom contacts detected by MAPIYA[9], upper distance cut-off 5 Å. The blue scale is decoded in the right panel.

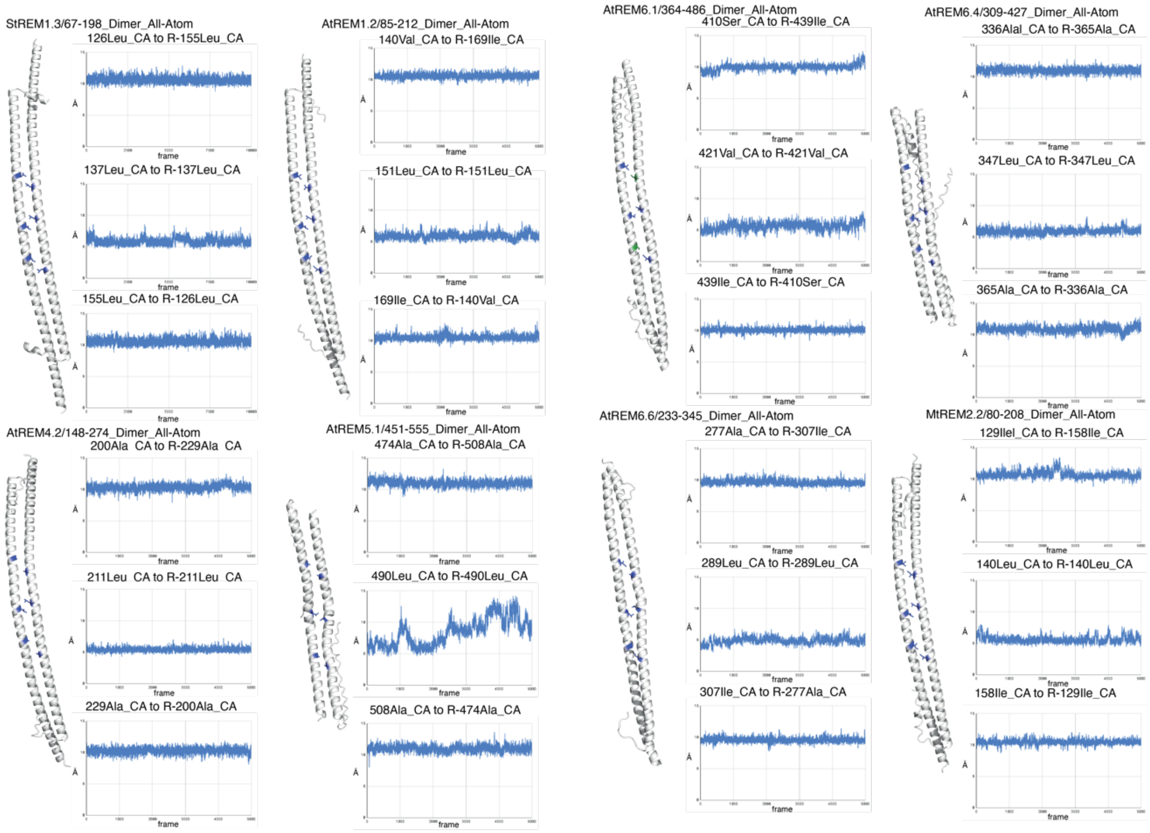

Supplementary Figure 15: Distance plot of the Cα-Cα contacts of the REM C-terminal region over 1 µs atomistic MD simulation of the aligned residues of the three selected residues of StREM$_{67-198}$ (Leu126$_{monomer_1}$-Leu155$_{monomer_2}$, Leu137$_{monomer_1}$-Leu137$_{monomer_2}$ and Leu155$_{monomer_1}$-Leu126$_{monomer_2}$), highlighted on the structure in the left panel.

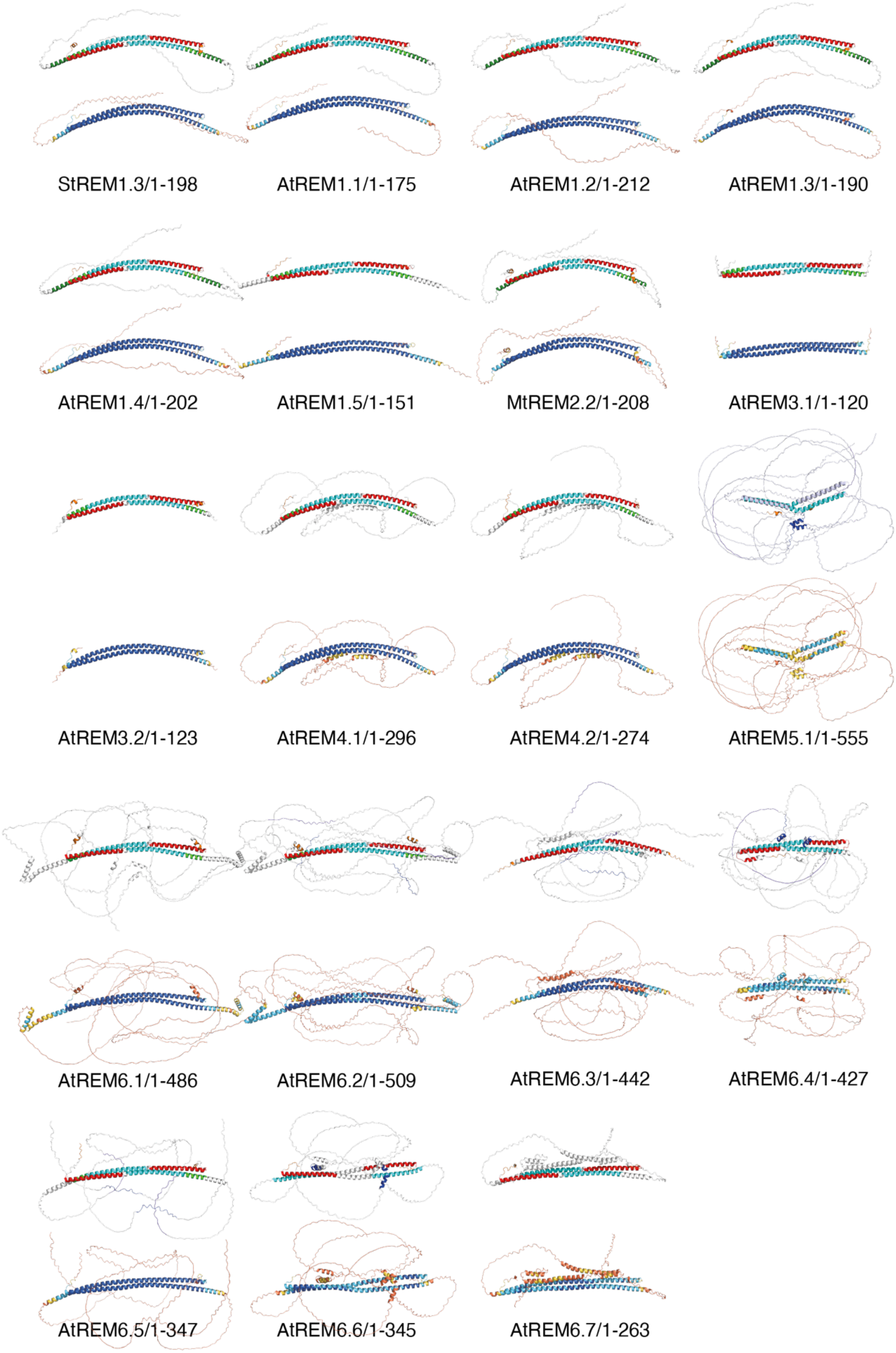

Supplementary Figure 16: One representative structure of AF2 dimer predictions for the REM family members. Structures are colored as the conserved sequence motifs 1 and 2 (detailed

in Figure 1 in the main text) in the upper panel and dependent on the pLDDT score (blue: pLDDT > 90; cyan 90 > pLDDT > 70; yellow 70 > pLDDT > 50; red pLDDT < 50, see also Figure 1 in the main text) in the lower panel, for each structure.

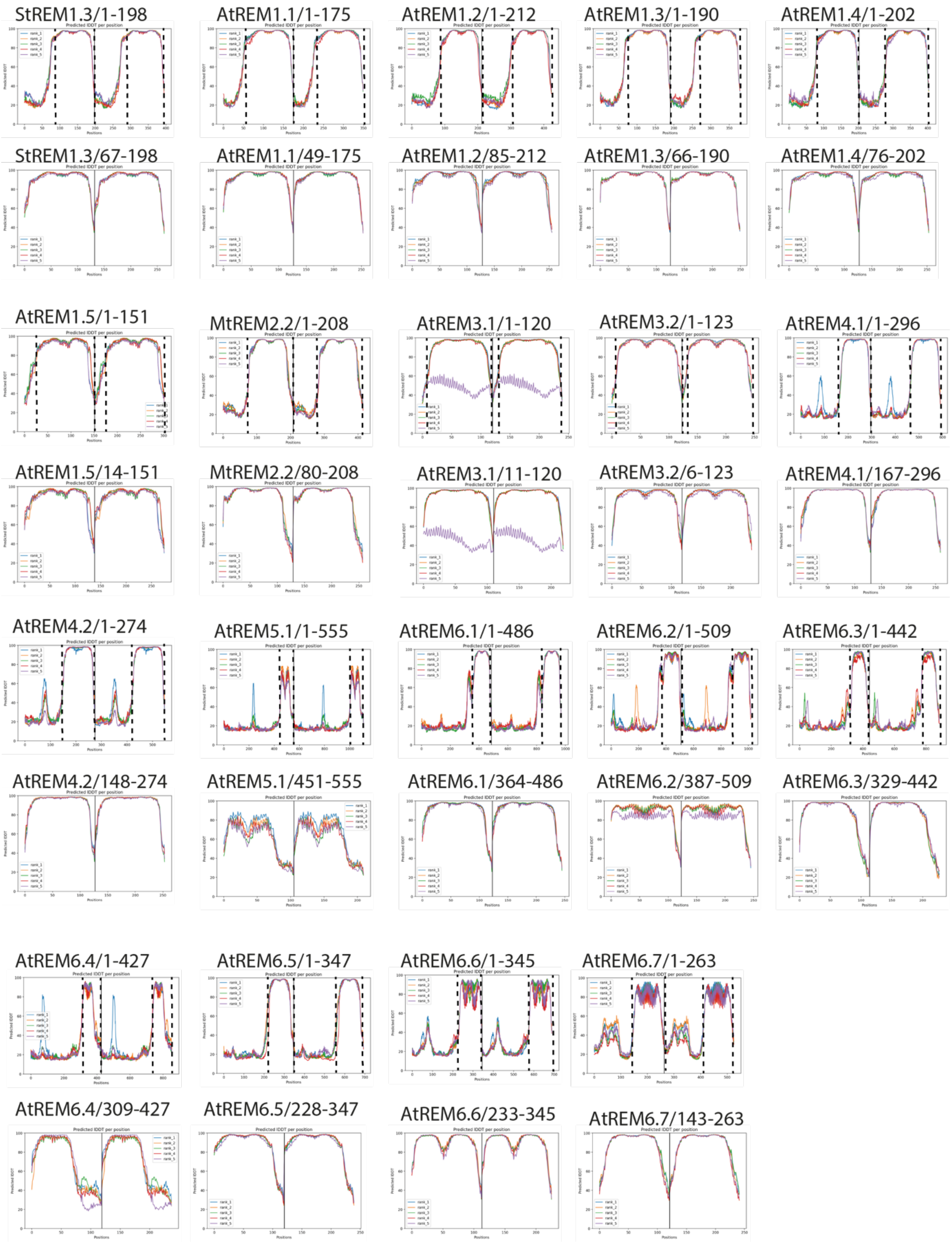

Supplementary Figure 17: AF2 pLDDT scores of the two monomers in the predicted dimeric coiled-coil structures plotted over the primary sequence. For each REM dimer the pLDDT

scores of five dimers are shown for the full-length REM (upper panel) and C-terminal region (lower panel).

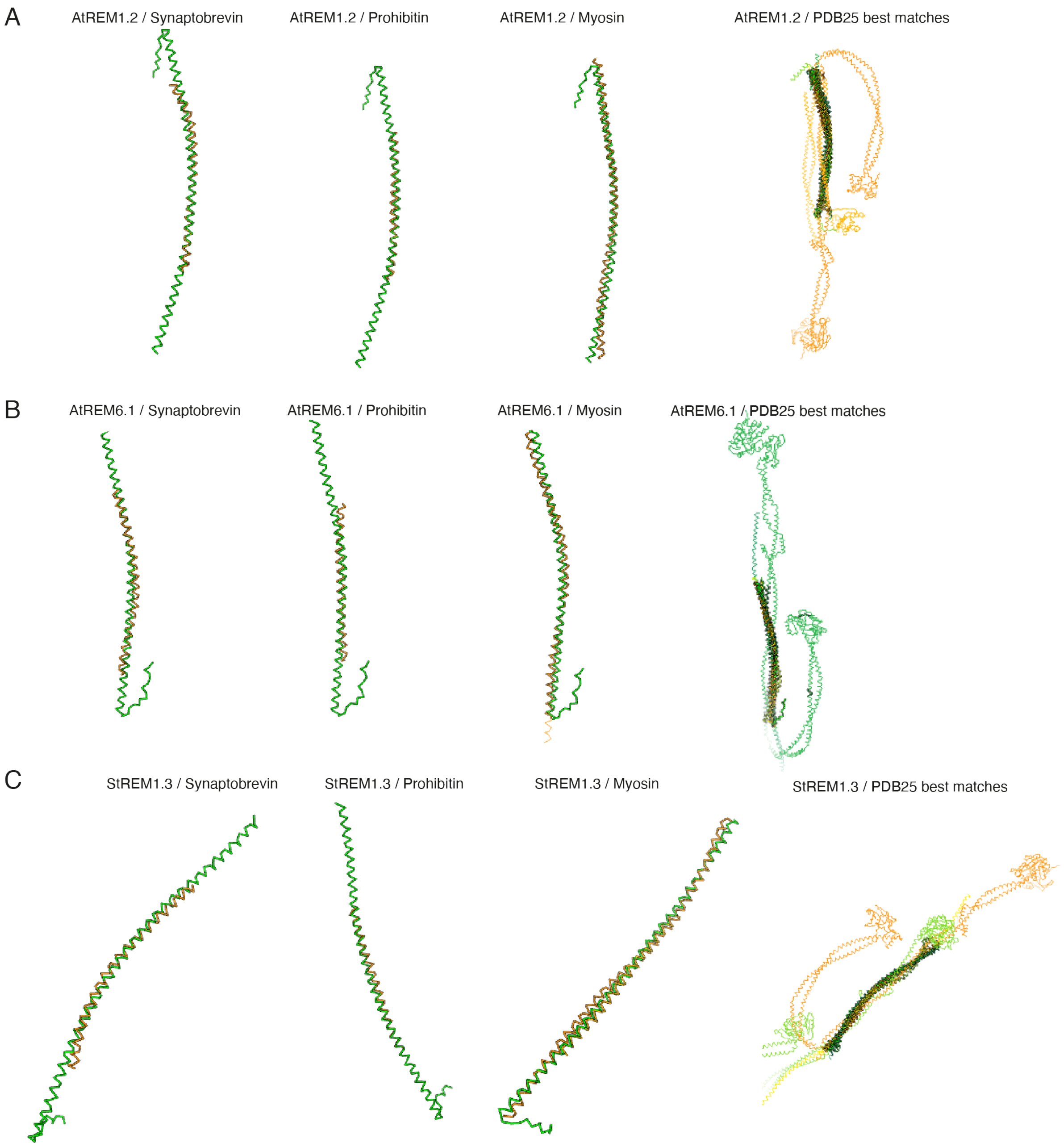

Supplementary Figure 18: Structural homology detected by Dali[10] for the C-terminal region of **a** AtREM1.2, **b** AtREM6.1 and **c** StREM1.3. Three selected monomeric structures of the restricted PDB25 set are shown aligned with REM C-terminal regions. The right panel shows the ten best aligned structures of the Dali[10] PDB25 set to the C-terminal regions of the respective protein.

References:

1. Raffaele, S., Mongrand, S., Gamas, P., Niebel, A. & Ott, T. Genome-Wide Annotation of Remorins, a Plant-Specific Protein Family: Evolutionary and Functional Perspectives. *Plant Physiol.* **145**, 593–600 (2007).
2. Bailey, T. L., Johnson, J., Grant, C. E. & Noble, W. S. The MEME Suite. *Nucleic Acids Res.* **43**, W39–W49 (2015).
3. Sievers, F. *et al.* Fast, scalable generation of high-quality protein multiple sequence alignments using Clustal Omega. *Mol. Syst. Biol.* **7**, 539 (2011).
4. Altschul, S. F., Gish, W., Miller, W., Myers, E. W. & Lipman, D. J. Basic local alignment search tool. *J. Mol. Biol.* **215**, 403–410 (1990).
5. Jumper, J. *et al.* Highly accurate protein structure prediction with AlphaFold. *Nature* **596**, 583–589 (2021).
6. Wang, Y. & Jardetzky, O. Probability-based protein secondary structure identification using combined NMR chemical-shift data. *Protein Sci* **11**, 852–61 (2002).
7. Brünger, A. T. *et al.* Crystallography & NMR System: A New Software Suite for Macromolecular Structure Determination. *Acta Crystallogr. D Biol. Crystallogr.* **54**, 905–921 (1998).
8. Case, D. A. *et al.* The Amber biomolecular simulation programs. *J. Comput. Chem.* **26**, 1668–1688 (2005).
9. Badaczewska-Dawid, A. E., Nithin, C., Wroblewski, K., Kurcinski, M. & Kmiecik, S. MAPIYA contact map server for identification and visualization of molecular interactions in proteins and biological complexes. *Nucleic Acids Res.* **50**, W474–W482 (2022).
10. Holm, L., Laiho, A., Törönen, P. & Salgado, M. DALI shines a light on remote homologs: One hundred discoveries. *Protein Sci.* **32**, e4519 (2023).
