# Supplementary material for: Dynamic pre-structuration of lipid nanodomain-segregating remorin proteins
Source: Commun Biol. 2024 Dec 5;7:1620. doi: 10.1038/s42003-024-07330-y (PMC11621693; doi:10.1038/s42003-024-07330-y)
Supplement: Supplementary file 2 — Description of Additional Supplementary Materials [file 42003_2024_7330_MOESM2_ESM.pdf]

Description of Additional Supplementary Files

File name: Supplementary Data 1

Description: primary sequences of remorin constructs used; detailed NMR restraint table; NMR restraints summary used for structure calculation

File name: Supplementary Data 2

Description: Dali predictions

File name: Supplementary Data 3

Description: Coconut predictions

File name: Supplementary Data 4

Description: The source data behind figures and graphs in the paper

File name: Supplementary Videos

Description: MD simulations using Amoeba FF
