# Supplementary material for: Dynamic pre-structuration of lipid nanodomain-segregating remorin proteins
Source: Commun Biol. 2024 Dec 5;7:1620. doi: 10.1038/s42003-024-07330-y (PMC11621693; doi:10.1038/s42003-024-07330-y)
Supplement: Supplementary file 5 — Supplementary Data 3 [file 42003_2024_7330_MOESM5_ESM.pdf]

AtREM1.1

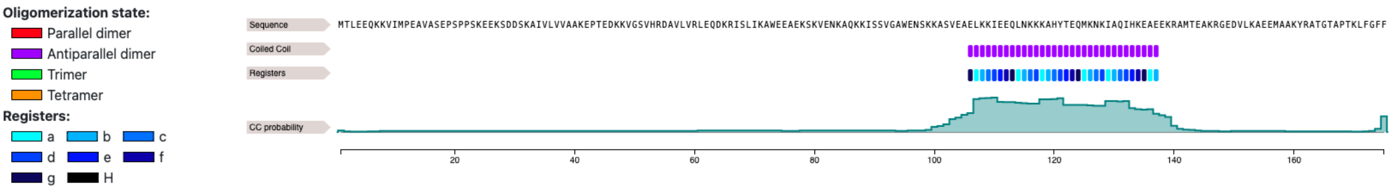

AtREM1.2

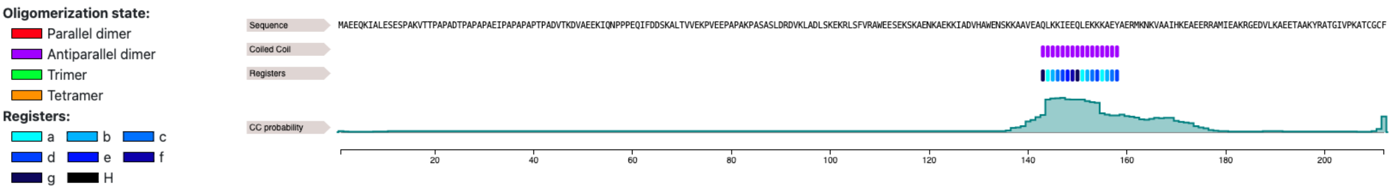

AtREM1.3

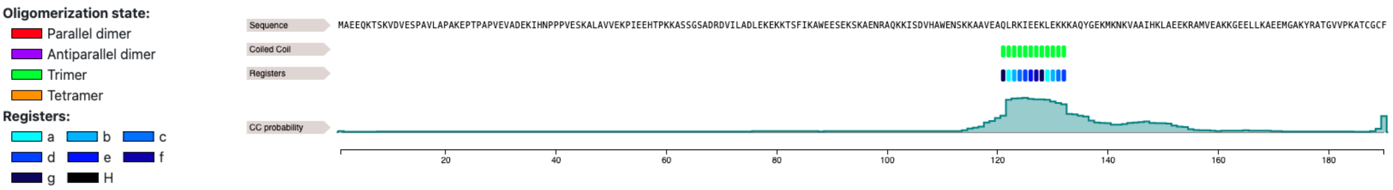

AtREM1.4

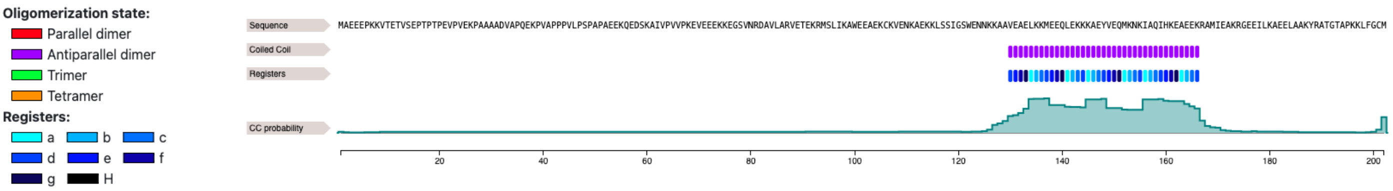

AtREM1.5

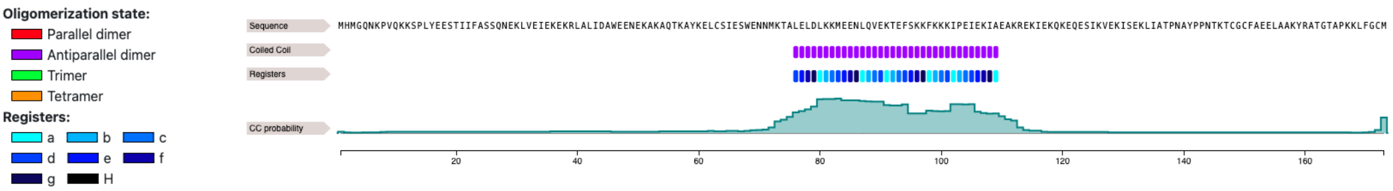

AtREM3.1

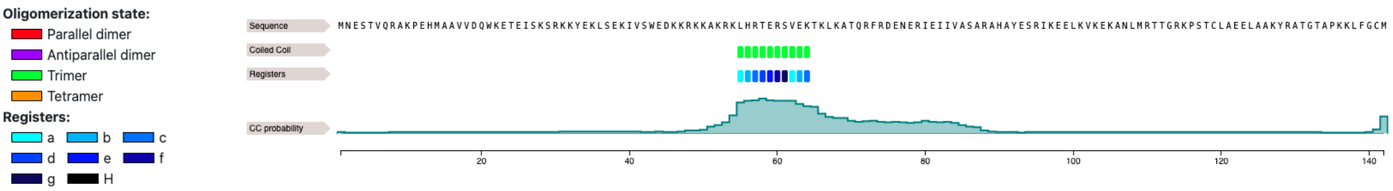

AtREM3.2

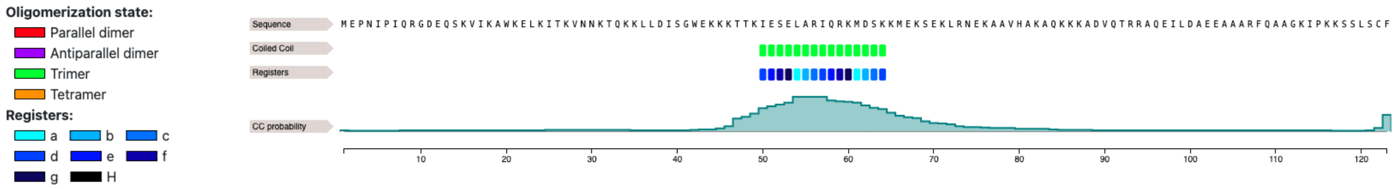

AtREM4.1

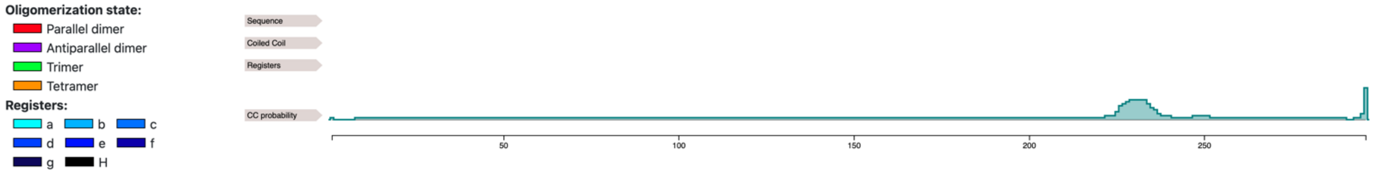

AtREM4.1 Zoom

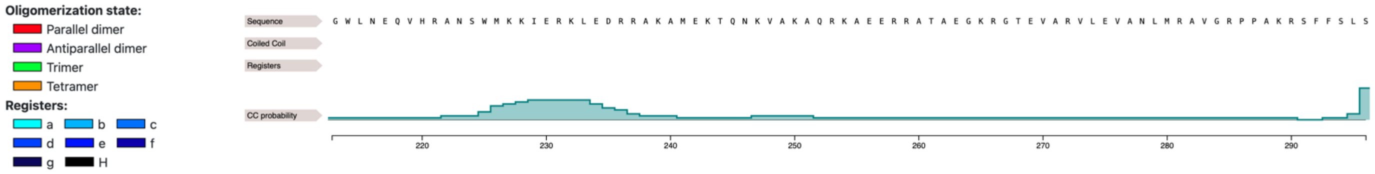

AtREM4.2

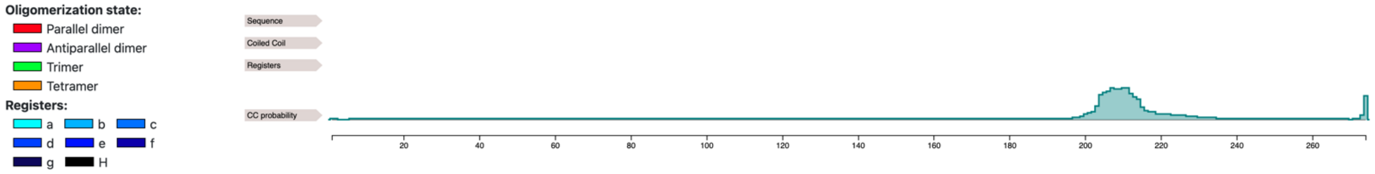

AtREM4.2 Zoom

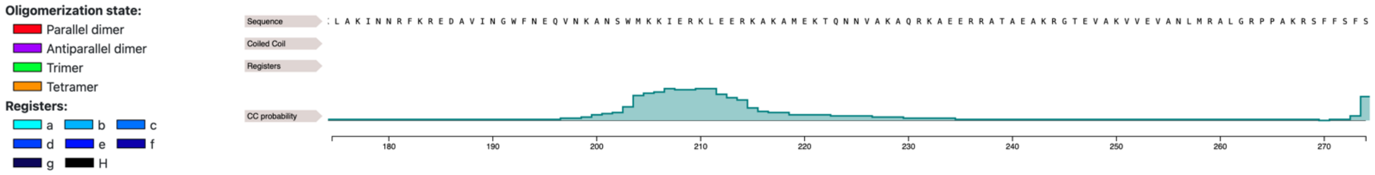

AtREM5.1

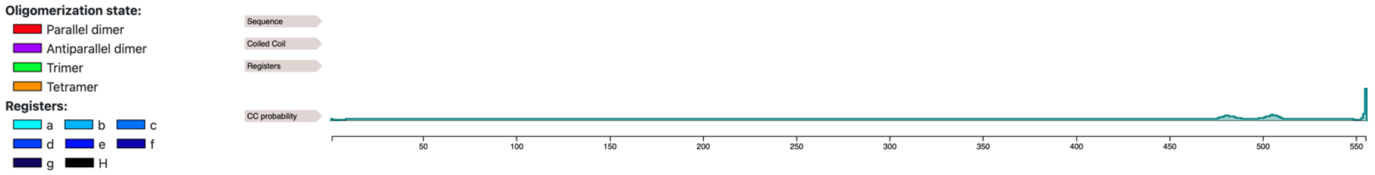

AtREM5.1 Zoom

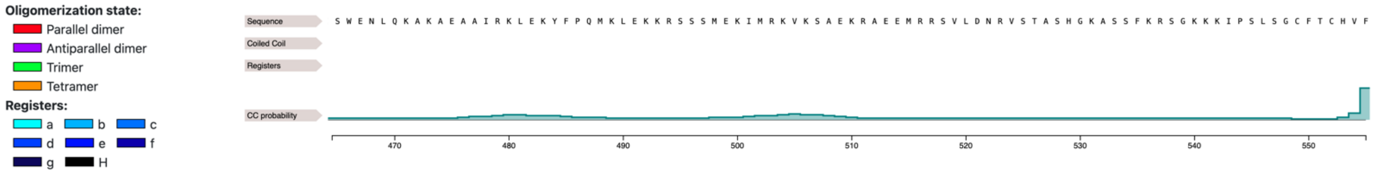

AtREM6.1

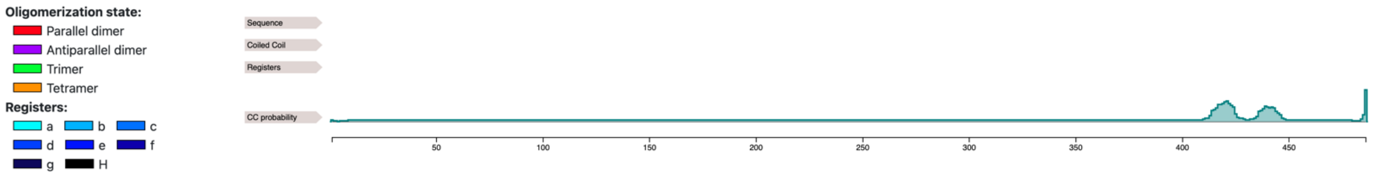

AtREM6.1 Zoom

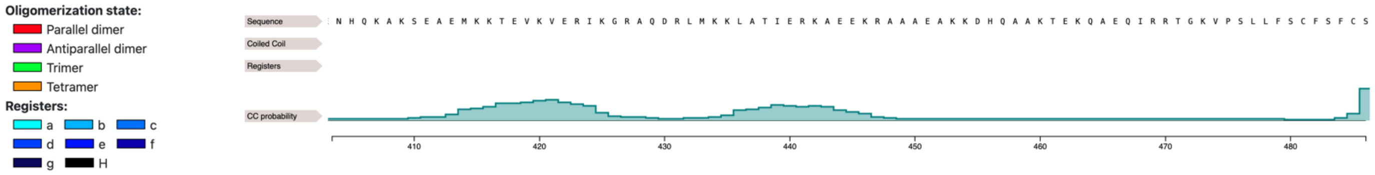

AtREM6.2

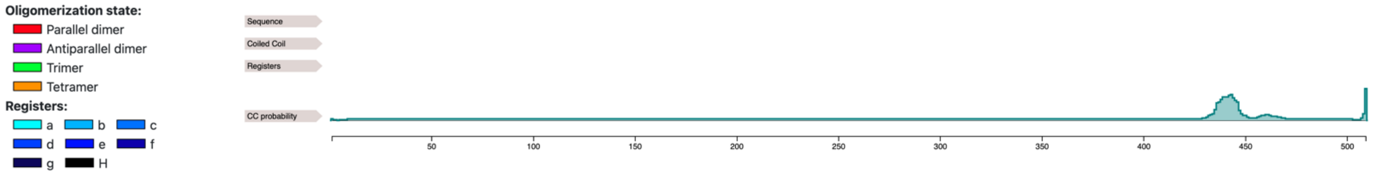

AtREM 6.2 Zoom

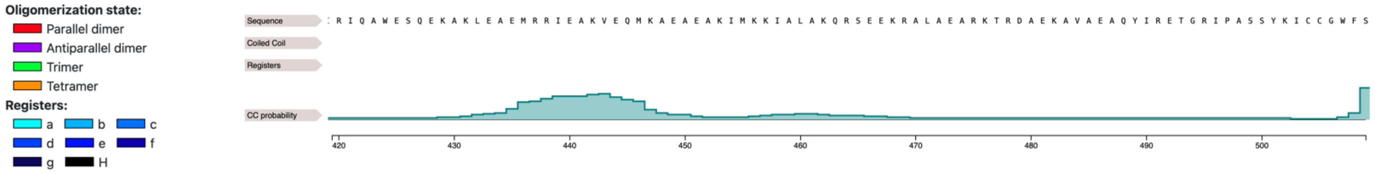

AtREM6.3

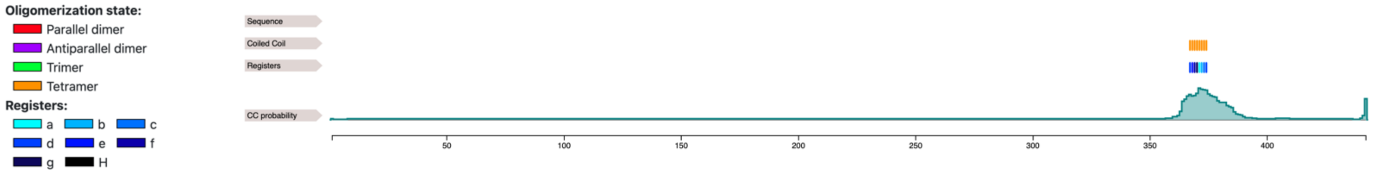

AtREM6.3 Zoom

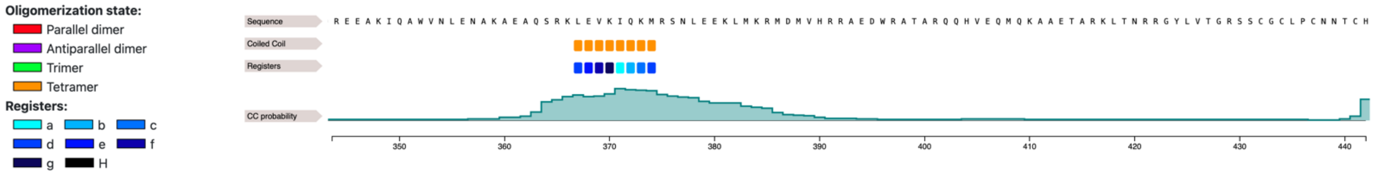

AtREM6.4

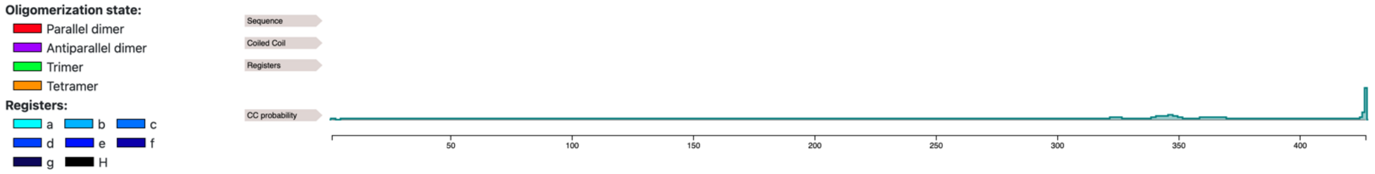

AtREM6.4 Zoom

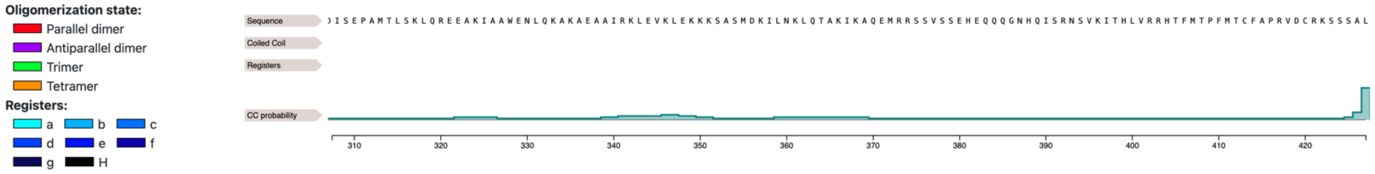

AtREM6.5

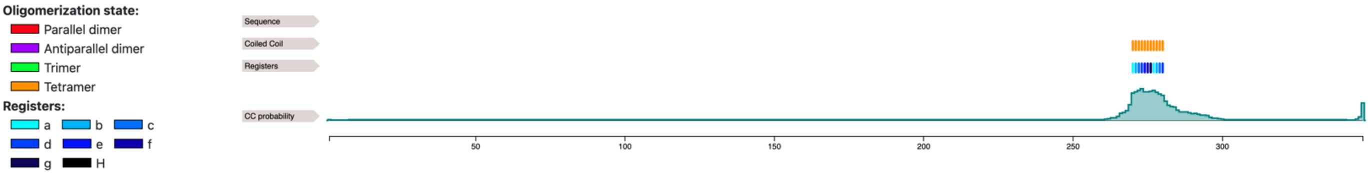

AtREM6.5 Zoom

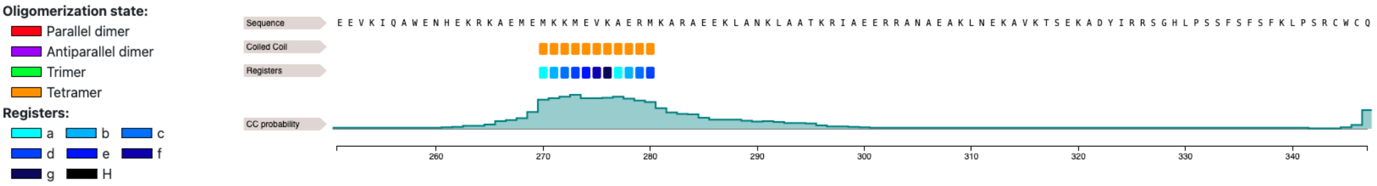

AtREM6.6

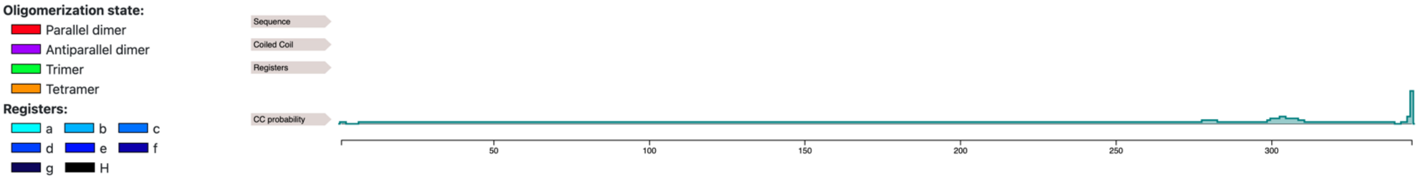

AtREM6.6 Zoom

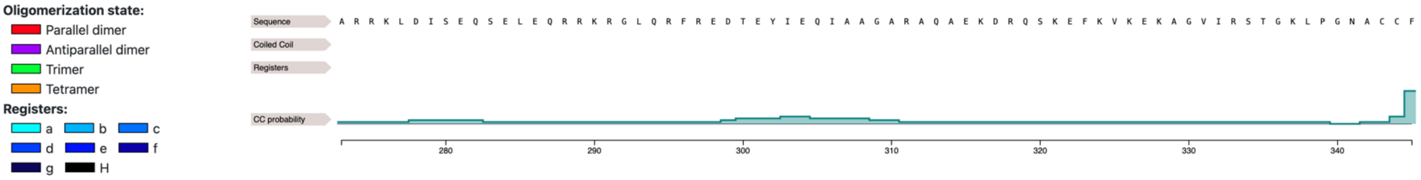

AtREM6.7

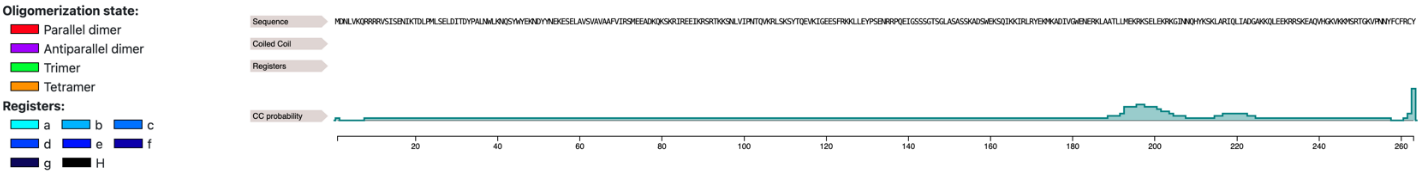

Supplementary Data: Coiled-coil oligomerization predicted by Coconat for the full-length REM proteins of several proteins from all REM groups (1-6). Color coding of the multimeric state and the register of the coiled-coil helical wheel is explained in the panel aside.
