# Supplementary material for: Dynamic pre-structuration of lipid nanodomain-segregating remorin proteins
Source: Commun Biol. 2024 Dec 5;7:1620. doi: 10.1038/s42003-024-07330-y (PMC11621693; doi:10.1038/s42003-024-07330-y)
Supplement: Supplementary file 8 — reporting summary [file 42003_2024_7330_MOESM8_ESM.pdf]

nature portfolio

Corresponding author(s): Matthieu Chavent
Sébastien Mongrand
Birgit Habenstein

Last updated by author(s): Nov 11, 2024

Reporting Summary

Nature Portfolio wishes to improve the reproducibility of the work that we publish. This form provides structure for consistency and transparency in reporting. For further information on Nature Portfolio policies, see our Editorial Policies and the Editorial Policy Checklist.

Statistics

For all statistical analyses, confirm that the following items are present in the figure legend, table legend, main text, or Methods section.

n/a	Confirmed	
☒	☐	The exact sample size (*n*) for each experimental group/condition, given as a discrete number and unit of measurement
☒	☐	A statement on whether measurements were taken from distinct samples or whether the same sample was measured repeatedly
☒	☐	The statistical test(s) used AND whether they are one- or two-sided<br>*Only common tests should be described solely by name; describe more complex techniques in the Methods section.*
☒	☐	A description of all covariates tested
☐	☒	A description of any assumptions or corrections, such as tests of normality and adjustment for multiple comparisons
☐	☒	A full description of the statistical parameters including central tendency (e.g. means) or other basic estimates (e.g. regression coefficient) AND variation (e.g. standard deviation) or associated estimates of uncertainty (e.g. confidence intervals)
☐	☒	For null hypothesis testing, the test statistic (e.g. *F*, *t*, *r*) with confidence intervals, effect sizes, degrees of freedom and *P* value noted<br>*Give P values as exact values whenever suitable.*
☒	☐	For Bayesian analysis, information on the choice of priors and Markov chain Monte Carlo settings
☒	☐	For hierarchical and complex designs, identification of the appropriate level for tests and full reporting of outcomes
☐	☒	Estimates of effect sizes (e.g. Cohen's *d*, Pearson's *r*), indicating how they were calculated

Our web collection on statistics for biologists contains articles on many of the points above.

Software and code

Policy information about availability of computer code

Data collection	Bruker Topspin 4.1;MEME Suite version 5.5.2; Amber ff99sb; Amber ff14sb; GROMACS; AMOEBA; PROPKA3; Tinker HP
Data analysis	Data Availability Statement<br>Software and code: CcpNMR Analysis 2.5.2 for NMR data analysis; CNS for structure calculation; Aria 2.0 for structure refinement; Multicoil2 for prediction of coiled-coil structure; Fiji using the Coloc2 plug-in; R (cran.r-project.org) Prism 6.0 for statistical analysis; Simulations were analysed using tcl code (available under https://doi.org/10.5281/zenodo.14163696) and the VMD software.

For manuscripts utilizing custom algorithms or software that are central to the research but not yet described in published literature, software must be made available to editors and reviewers. We strongly encourage code deposition in a community repository (e.g. GitHub). See the Nature Portfolio guidelines for submitting code & software for further information.

Data

Policy information about availability of data

All manuscripts must include a data availability statement. This statement should provide the following information, where applicable:

- Accession codes, unique identifiers, or web links for publicly available datasets
- A description of any restrictions on data availability
- For clinical datasets or third party data, please ensure that the statement adheres to our policy

Chemical shifts have been deposited under the following accession codes StREM13_160-198 BMRB ID: 52390; StREM13_171-198 BMRB ID: 52391; StREM13_150-198 BMRB ID: 52393; AtREM11_156-175 BMRB ID: 52402; AtREM12_193-212 BMRB ID: 52403; AtREM13_171-190 BMRB ID: 52404; MtREM22_189-208 BMRB ID: 52405; AtREM41_277-296 BMRB ID: 52406; AtREM51_536-555 BMRB ID: 52407; AtREM62_490-509 BMRB ID: 52408; AtREM63_423-442 BMRB ID: 52409; AtREM64_408-427 BMRB ID: 52410; AtREM65_328-347 BMRB ID: 52411. Structural ensembles have been deposited for StREM1.3171-198, StREM160-198 and StREM150-198 under the following identifiers PDB ID 9F1E, PDB ID 9F1F and PDB ID 9F1G, respectively. Primary sequences of remorins used, detailed NMR restraints have been deposited in supplementary data 1. Data used to create the Figures have been deposited in the Supplementary data 4. MD simulation data have been deposited in Zenodo under the following access: https://doi.org/10.5281/zenodo.14163696

Human research participants

Policy information about studies involving human research participants and Sex and Gender in Research.

Reporting on sex and gender	NA
Population characteristics	NA
Recruitment	NA
Ethics oversight	NA

Note that full information on the approval of the study protocol must also be provided in the manuscript.

Field-specific reporting

Please select the one below that is the best fit for your research. If you are not sure, read the appropriate sections before making your selection.

☒ Life sciences ☐ Behavioural & social sciences ☐ Ecological, evolutionary & environmental sciences

For a reference copy of the document with all sections, see nature.com/documents/nr-reporting-summary-flat.pdf

Life sciences study design

All studies must disclose on these points even when the disclosure is negative.

Sample size	Nicotiana Benthamiana leaves were aagroinfiltrated to transform cells with fluroescent tagged proteins, At least 23 cells were analyzed for each condition over the course of 3-4 experiments.
Data exclusions	no data exclusion
Replication	All attempts of replication were successful.
Randomization	no randomization
Blinding	no blinding

Reporting for specific materials, systems and methods

We require information from authors about some types of materials, experimental systems and methods used in many studies. Here, indicate whether each material, system or method listed is relevant to your study. If you are not sure if a list item applies to your research, read the appropriate section before selecting a response.

Materials & experimental systems

n/a	Involved in the study
☒	☐ Antibodies
☒	☐ Eukaryotic cell lines
☒	☐ Palaeontology and archaeology
☒	☐ Animals and other organisms
☒	☐ Clinical data
☐	☒ Dual use research of concern

Methods

n/a	Involved in the study
☒	☐ ChIP-seq
☒	☐ Flow cytometry
☒	☐ MRI-based neuroimaging

Dual use research of concern

Policy information about [dual use research of concern]

Hazards

Could the accidental, deliberate or reckless misuse of agents or technologies generated in the work, or the application of information presented in the manuscript, pose a threat to:

No	Yes
☒	☐ Public health
☒	☐ National security
☒	☐ Crops and/or livestock
☒	☐ Ecosystems
☒	☐ Any other significant area

Experiments of concern

Does the work involve any of these experiments of concern:

No	Yes
☒	☐ Demonstrate how to render a vaccine ineffective
☒	☐ Confer resistance to therapeutically useful antibiotics or antiviral agents
☒	☐ Enhance the virulence of a pathogen or render a nonpathogen virulent
☒	☐ Increase transmissibility of a pathogen
☒	☐ Alter the host range of a pathogen
☒	☐ Enable evasion of diagnostic/detection modalities
☒	☐ Enable the weaponization of a biological agent or toxin
☒	☐ Any other potentially harmful combination of experiments and agents
